# Anteroposterior Wnt-RA Gradient Defines Adhesion and Migration Properties of Neural Progenitors in Developing Spinal Cord

**DOI:** 10.1016/j.stemcr.2020.08.016

**Published:** 2020-09-24

**Authors:** Mohammed R. Shaker, Ju-Hyun Lee, Si-Hyung Park, Joo Yeon Kim, Gi Hoon Son, Jong Wan Son, Bae Ho Park, Im Joo Rhyu, Hyun Kim, Woong Sun

**Affiliations:** 1 Department of Anatomy, Brain Korea 21 Plus Program, Korea University College of Medicine, Seoul, 02841, Korea; 2 Australian Institute for Bioengineering and Nanotechnology (AIBN), The University of Queensland, St. Lucia, Brisbane, QLD 4072, Australia; 3 Department of Legal Medicine, College of Medicine, Korea University, Seoul 02841, Korea; 4 Department of Biomedical Sciences, College of Medicine, Korea University, Seoul 02841, Korea; 5 Division of Quantum Phases and Devices, Department of Physics, Konkuk University, Seoul 05029, Korea

**Keywords:** neural stem cells, axial elongation, adhesion and migration, neuromesodermal progenitors, Wnt-RA network, extracellular matrix

## Abstract

Mammalian embryos exhibit a transition from head morphogenesis to trunk elongation to meet the demand of axial elongation. The caudal neural tube (NT) is formed with neural progenitors (NPCs) derived from neuromesodermal progenitors localized at the tail tip. However, the molecular and cellular basis of elongating NT morphogenesis is yet elusive. Here, we provide evidence that caudal NPCs exhibit strong adhesion affinity that is gradually decreased along the anteroposterior (AP) axis in mouse embryonic spinal cord and human cellular models. Strong cell-cell adhesion causes collective migration, allowing AP alignment of NPCs depending on their birthdate. We further validated that this axial adhesion gradient is associated with the extracellular matrix and is under the control of graded Wnt signaling emanating from tail buds and antagonistic retinoic acid (RA) signaling. These results suggest that progressive reduction of NPC adhesion along the AP axis is under the control of Wnt-RA molecular networks, which is essential for a proper elongation of the spinal cord.

## Highlights

• NPCs along the spinal cord exhibit a gradient of cell adhesion (anterior < posterior)
• Cell adhesion changes depending on the position and developmental stage of NPCs
• Wnt-RA signaling acts as an upstream effector of NPCs
• Microarray analysis revealed enrichment of ECM among posterior NPCs

## Graphical Abstract

**Figure:**
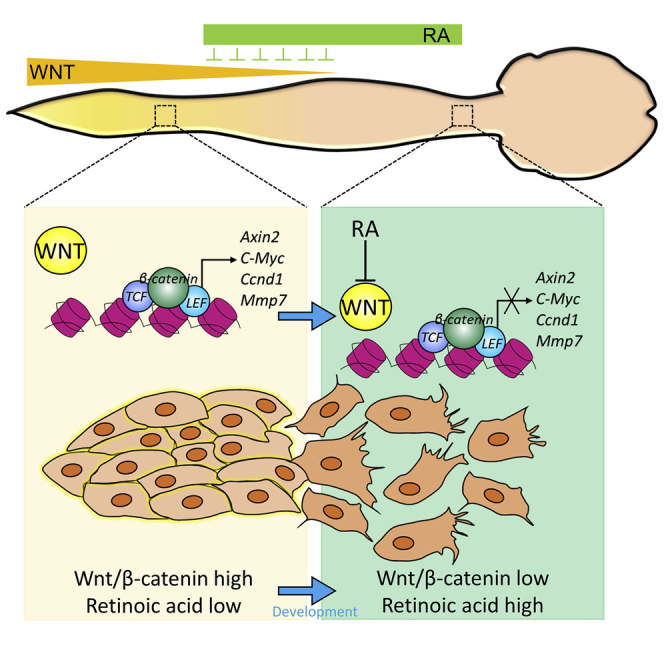

## Teaser

In this article, Shaker and colleagues show that cell-cell adhesion and cell-migration properties of the NPCs in the elongating embryonic spinal cord were different depending on their anteroposterior position (anterior < posterior). Transcriptomic analysis revealed ECM enrichment in posterior NPCs, which is dependent on anteroposterior gradient of Wnt-RA signaling.

## Introduction

The vertebrate central nervous system (CNS) is organized along the anteroposterior (AP) axis. At the anterior level, the neuroectoderm is the first ancestor to emerge during CNS development. The neuroectoderm switches to neuroepithelial cells (NECs), which generate a large pool of neural stem cells (NSCs) and eventually produce most CNS cell types, including neurons, astrocytes, oligodendrocytes, and ependymal cells. Morphologically, NECs first form the neural plate, which subsequently undergoes convergent extension, elevation, bending, adhesion, and fusion to form the neural tube (NT), a primitive structure of the CNS (Pai et al., 2012). These early neurulation events produce the anterior part of the body, whereas a transition occurs at the tail-bud level to meet the demand for the posterior body axis elongation, where biopotent neuromesodermal progenitors (NMPs) continually provide neural tissues (Henrique et al., 2015). While NMPs provide trunk neural tissues before neurulation and can contribute to primary neurulation, axial elongation continues after trunk elongation and a distinct developmental morphogenetic event for extending the caudal part of the trunk and tail (Steventon and Arias, 2017). These caudal elongations differ from the early phase with respect to the underlying mechanisms that rely less on the convergence extension and ingression of NECs. This second phase of morphogenesis is often defined as secondary neurulation, which includes aggregation, cavitation, and caudal-to-rostral migration of NMP-derived neural progenitors (NPCs) (Kawachi et al., 2020).

The NPCs in the NT are, thus, roughly divided into two groups depending on their developmental origin and position, namely, anterior NE-derived NPCs and posterior-NMP-derived NPCs. We have addressed the regional differences in NPCs and reported that NPCs from the posterior NT lose their identity and differentiate faster even in the presence of epidermal growth factor and basic fibroblast growth factor (Shaker et al., 2015). Consistent with our findings, the developing human spinal cord-derived neurospheres have limited life span and neurogenic potential compared with those derived from the brain (Kukekov et al., 1997). Studies involving two different culture approaches showed that the human spinal cord contains different neural precursors (Walder and Ferretti, 2004). In addition, several mutations affect the formation of the spinal axis but not brain structures (Handrigan, 2003). Thus, it is obvious that not all NPCs are equivalent, and the differences in their properties are attributed to their developmental origin and final environment.

It is well established that local morphogen gradients are implicated in NT patterning. Caudal factors such as *Wnts*, *Gdf11*, and *Fgfs* are expressed at the tail bud of the developing spinal cord, and inhibition of them arrests trunk elongation (Young et al., 2009). On the other hand, retinoic acid (RA) is secreted by anteriorly positioned somites and defines the anterior identity by suppressing caudal Wnt and fibroblast growth factor (FGF) signaling (Tenin et al., 2010). FGFs and Wnts also regulate axial elongation at least partly by affecting the proliferative pool size of NMPs. Hence, different NPCs along the AP axis of the developing spinal cord are exposed to different-graded signaling, which in turn defines the positional identity with the expression of distinct *Hox* (Zheng et al., 2015). Although cellular heterogeneity and gradient signals for AP patterning are relatively well described, how these regional differences in NPC identity and environmental signals contribute to caudal NT elongation and morphogenesis is less understood.

Thus, it is obvious that not all NPCs are equivalent, and the differences in their properties are attributed to their developmental origin and final environment. Axial elongation involves morphological changes in cells and their movements. For somitogenesis, caudal mesoderm progenitor cells increase in their protrusive activity, rotate to align along the mediolateral axis, and migrate rostrally to build up the somites (Afonin et al., 2006). Neural tissues are also highly polarized with strong adherens, gap, and tight junctions (Shimokita and Takahashi, 2011). After the NT is formed, NSC-derived NPCs start migrating radially or tangentially out of the NT, which is partially regulated by cell adhesion, resulting in various morphological changes. Therefore, it is presumable that dynamic alterations in the adhesion and migration of NPCs is required for the caudal elongation and NT morphogenesis. In fact, the differential interfacial tension hypothesis (DITH) is the best-known theory that explains the spontaneous cell-cell interactions and patterning of embryos during development (Brodland, 2002). This mathematical model postulates that adhesive cells sort to the periphery and envelop the stronger adhesive cells, which is based on the differences in cortical tension driven by the actomyosin cytoskeleton. Eventually, many studies applied this model to study the adhesion properties of several cell types (Canty et al., 2017; Stirbat et al., 2013). In this study, we attempted to gain a detailed understating of the cellular behavior of NPCs during NT elongation, and accordingly, we addressed (1) the difference in the adhesion of NPCs depending on the position in the AP axis; (2) the difference in NPC adhesion promotes distinct migration behaviors; and (3) whether the mechanism mediating NPC behavior in AP gradients is morphogen dependent. Based on our exploration, we propose that caudal Wnt signaling control the adhesive and migratory properties of NPCs, which is dependent by the caudal secretion of RA.

## Results

### Differential Cell Adhesion Properties of NPCs along the AP Axis of the Developing NT

**Figure 1:**
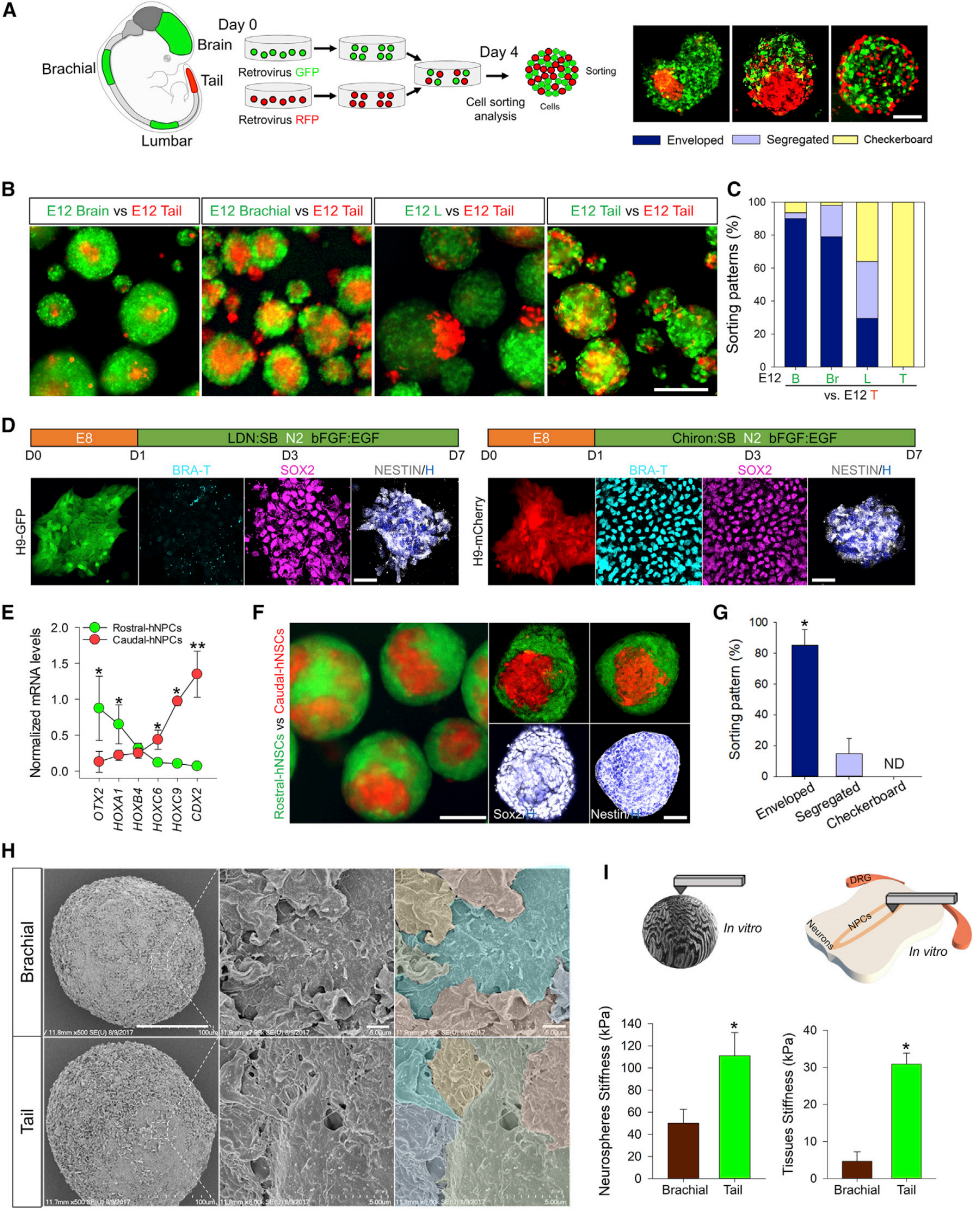
Evidence of Strong Cell-Cell Adhesion among Caudal NPCs (A) Schematic diagram of E12 embryo primitive CNS domains (the neural tube) and diagram of cell-sorting assay where cells self-sort based on their adhesion affinity. The right shows confocal images of sorted cryosectioned neurospheres. Scale bar, 75 μm. (B) Representative images of co-cultured NPC re-aggregates. NPCs at different times and positions were marked with GFP or RFP. E12 brain (GFP), E12 brachial (GFP), E12 lumbar (GFP), and E12 tail (RFP/GFP). Scale bar, 200 μm. (C) Quantification of the percentage of sorting patterns. Brain (B) versus tail (T) resulted in significantly (90%) higher enveloped phenotype than segregated 3.5% and checkerboard 6.5%; brachial (Br) versus T resulted in significantly (78.2%) higher enveloped phenotype than segregated 19.65% and checkerboard 2% phenotypes; lumbar (L) versus T resulted in 29% enveloped, 34.5% segregated, and 36% checkerboard; T versus T resulted in 100% checkerboard phenotype. Data are shown as percentage; number of independent experiments = 4; number of examined neurospheres = 705. (D) Schematic diagram of the experimental procedure for generation of rostral and caudal NPCs. Color-coded H9 hESCs were differentiated directly to rostral NPCs via neuroectoderm stage or caudal NPCs via the NMP stage. Images represent serial stages of differentiation of colony morphology stained with SOX2 (magenta), BRA-T (cyan), and NESTIN (gray). Nuclei were counterstained with Hoechst 33342 (blue). Scale bar, 15 μm. (E) qRT-PCR verification of AP regional identity using various *Hox* gene expressions. Data are shown as mean ± SD; number of independent experiments = 3; ^∗^p < 0.05; ^∗∗^p < 0.001. (F) Representative images of re-aggregates of rostral (green) and caudal (red) NPCs for 4 days (left). Images on the right show the whole-mount labeling with SOX2 (gray) and NESTIN (gray). Nuclei were counterstained with Hoechst 33342 (blue). Scale bar, 150 μm for low-magnification image. Scale bar, 75 μm for high-magnification images. (G) Quantification of the percentage of sorting phenotypes in (C). Data are shown as mean ± SD; number of independent experiments = 3; number of examined neurospheres = 260; ^∗^p < 0.001 via one-way ANOVA. ND, not detected. (H) Scanning electron microscope images of neurospheres. Left images show entire neurospheres and the magnified images of dotted squares are shown in the middle. Images with the pseudo-coloring of each cell are on the right. Left image scale bar, 100 μm. Magnified image scale bar, 5 μm. The number of examined neurospheres = 12. (I) Measurements of tissue stiffness via AFM. Upper images show the schematic diagram of AFM measurement. Lower graphs show the measurement of surface stiffness of neurospheres (left) and ventricular zone of the NT (right). Data are shown as mean ± SD; ^∗^p < 0.001 via Student's t test; number of independent experiments = 3; number of examined fields = 25. Abbreviation: DRG, dorsal root ganglion.

We performed a cell-sorting assay to obtain the estimation of cell-cell adhesion of NPCs at different levels of the NT (Schötz et al., 2008). NPCs were isolated from different regions of the NT and expanded by neurosphere culture *in vitro* (Figures 1A, and S1A). Tail-derived NPCs were labeled with retrovirus-RFP and mixed with GFP-labeled NPCs derived from the brain or brachial level in a 1:1 ratio (Figure 1A). Re-aggregated neurospheres exhibited distinct sorting phenotypes, including checkerboard (cells have similar adhesion affinity), segregated (similar adhesion with weak interaction), and enveloped (core cells have stronger adhesion than periphery cells) (Figure 1A). Interestingly, we identified a strong gradient of cell-sorting phenotypes along the AP axis. A mixture of brain-derived and tail-derived NPCs resulted in >70% enveloped phenotype, with tail-derived NPCs in the core (Figures 1B and 1C). The percentage of enveloped-type re-aggregates was progressively reduced when tail-derived NPCs were mixed with NPCs from lower positions along the AP axis, with a compensatory increase in the segregated- and checkerboard-type re-aggregates. Expression of molecular markers for NPCs (SOX2 and NESTIN) and differentiation of these cells upon growth factor withdrawal yielded multiple types of neural cells, confirming that these are indeed NPCs irrespective of their adhesion properties (Figure S1B). A complete absence of *Bra-T*-expressing NMPs or mesodermal cells in the neurospheres was confirmed by RT-PCR and immunostaining (Figures S1B and S1C). We found the weak expression of *Snail1* and *Pax3* (markers for neural crest cells) in both brachial- and tail-derived NPCs (Figure S1C), and we failed to completely rule out the possibility that neural crest cells were not completely removed. However, it is unlikely that the potential contamination of neural crest cells significantly affected the experimental outcome or data interpretation.

We also tested whether human NPCs (hNPCs) demonstrated AP axis-dependent properties similar to those observed in mouse NPCs. Human H9 embryonic stem cells (hESCs) were differentiated into rostral or caudal NPCs using a published protocol (Lippmann et al., 2015) (Figure 1D), and their regional identity was validated with *HOX* expression patterns (Figure 1E). Similar to the results obtained with mouse NPCs, the majority of caudal hNPCs were enveloped by rostral hNPCs in the sorting assay (Figures 1F and 1G, and Video S1), suggesting that AP axis-dependent gradient of adhesion property is evolutionarily conserved in human and mouse.

Video S1. Re-aggregation of Rostral (GFP) and Caudal (mCherry) hNPCs Demonstrating the Enveloped Phenotype Cell re-aggregation was monitored using a Juli stage equipped with a 4× objective lens and placed in a 5% CO_2_ incubator at 37°C. Images were taken every 10 min for 48 h.

A comparison of scanning electron microscopy images of neurospheres from brachial and tail regions showed significant differences in the surface morphology (Figure 1H), as the surface of brachial-derived neurospheres was rough and cell margins were easily identifiable, whereas the surface of tail-derived neurospheres was rather smooth and the cell margins were difficult to identify (Figure 1H, individual cells are differentially color coded in magnified images on the right). A similar difference was also observed in neurospheres derived from rostral and caudal hNPCs (Figure S2A). To directly assess the physical strength of the neurosphere, we measured the stiffness of the neurospheres and embryonic NT with atomic force microscopy (AFM). Consistent with the sorting assay data, significantly higher stiffness was observed at the tail neurosphere/NT than at the brachial neurosphere/NT in both measurements (Figure 1I). Altogether, these data support the idea that cell-cell adhesion properties of NPCs are different depending on their original position along the AP axis of the developing spinal cord.

### Differential Adhesion Affinity of NPCs Is Associated with Developmental Stages within the Same NT Domain

**Figure 2:**
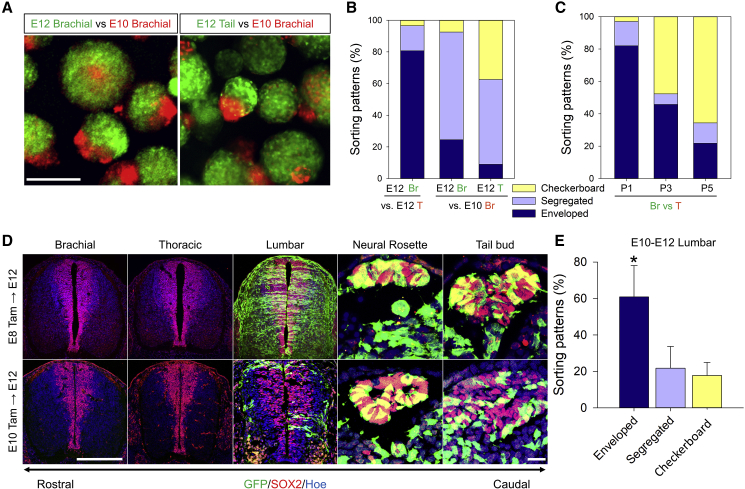
Cellular Adhesion of Caudal NPCs Is Birthdate Dependent (A) Representative images of NPC re-aggregates. E12 brachial (GFP), E12 tail (GFP), and E10 brachial (RFP). Scale bar, 200 μm. (B) Quantification of the percentage of sorting patterns. E12 Br versus E12 T resulted in significantly (80%) higher enveloped phenotype than segregated 16.5% and checkerboard 3.5% phenotypes; E12 Br versus E10 Br resulted in significantly (70%) higher segregated phenotype than enveloped 27% and checkerboard 3% phenotypes. E12 T versus E10 Br resulted in significantly (53.5%) higher segregated and (38%) checkerboard phenotypes than (9%) enveloped phenotypes. Data are shown as percentage; number of independent experiments = 3; number of examined neurospheres = 525. (C) Quantification of the percentage of sorting patterns upon passaging E12 NPCs. Br versus T-P1 resulted in significantly (82%) higher enveloped phenotype than segregated 15% and checkerboard 3% phenotypes; Br versus T-P3 resulted in significantly (45.5%) higher enveloped and (47.7%) checkerboard phenotypes than (6.5%) segregated phenotype; Br versus T-P5 resulted in significantly (65.6%) higher checkerboard phenotype than (21.7%) enveloped and (12.5%) segregated phenotypes. Data are shown as percentage; number of independent experiments = 4; number of examined neurospheres = 809. (D) Transverse sections of TCreERT2:Rosa-EGFP embryo following immunostaining with GFP (green) and SOX2 (red). Nuclei were counterstained with Hoechst 33342 (blue). Scale bars, 100 μm and 30 μm. The number of examined animals = 24. (E) Quantification of the percentage of sorting phenotypes in lumbar-derived neurospheres. Lumbar tissue was dissected out of E10–E12 TCreERT2:Rosa-EGFP embryos. Data are shown as mean ± SD; number of independent experiments = 3; number of examined neurospheres = 735; ^∗^p < 0.001 via one-way ANOVA. Abbreviation: E, mouse embryonic day.

NT development initiates from the rostral region, and the above-mentioned sorting phenotype gradients might be associated with the timing of NPC production depending on the AP axis. Supporting this idea, a significantly larger proportion of aggregates exhibited segregated and enveloped phenotypes in re-aggregation from the same domain (brachial) at different stages (E10 versus E12), with younger NPCs occupying the core region of the enveloped types (Figures 2A and 2B), suggesting that cell adhesion affinity is associated with birthdate and may decrease during the development. The mixture of the E10 brachial-derived NPCs and the E12 tail-derived NPCs resulted in a higher proportion of checkerboard and segregated phenotypes compared with the homochronic (i.e., E12 brachial and E12 tail NPCs) mixtures (Figure 2B). In addition, we passaged the brachial- and tail-derived NPCs and examined their cell-sorting properties *in vitro* (Figure 2C). By the passaging, the difference in cell adhesion affinity between two populations decreased, and accordingly, the enveloped phenotype was progressively reduced. These data may indicate that recently born, tail-derived NPCs progressively lose their strong adhesion properties during the proliferation/passage *in vitro*. Collectively, these data suggest that these adhesion properties are dynamically changing depending on the position and developmental stage of NPCs.

To address this issue precisely, we labeled and chased the NMP-derived NPCs by crossing transgenic TCreERT2 mice with Rosa-EGFP reporter mice followed by injecting tamoxifen (TAM) at E10, when caudal NPCs are produced and contribute to the lumbar NT. Because most of NPCs in the lumbar domain are generated from the earlier E8 NMPs (Figure 2D), we were able to identify GFP^+^ (late-borne) and GFP^−^ (early-borne) cells from the lumbar level with TAM injection at E10, and we explored their cell adhesion properties in the mixture. As expected, all neurospheres derived from brachial and tail levels were GFP^−^ and GFP^+^, respectively (Figure S2B), but neurospheres derived from lumbar levels were a mixture of GFP^+^ and GFP^−^ cells. All sorting phenotypes were found in the lumbar mixture (Figure S2, magnified images), with the enveloped phenotype representing the significant majority compared with the segregated and checkerboard phenotypes (Figure 2E). On the other hand, GFP^+^ cells in E12 brachial neurospheres labeled by E6 TAM injection exhibited mostly a checkerboard sorting phenotype (Figure S5C), suggesting that the birthdate-dependent differences in the adhesion properties are attenuated over time. Altogether, these data suggest that NPCs in a similar (i.e., lumbar) level of the NT also exhibit differential cell adhesion properties transiently depending on their birthdate.

Of note is that the GFP^+^ NPCs in the neural rosette and tail bud were not clearly segregated from the GFP^−^ NPCs *in vivo* (Figure 2D). We speculate that this is owing to the insufficient Cre recombination upon TAM treatment (Shaker et al., 2020), but we cannot completely rule out the possibility that the cell-sorting behavior of the NPCs *in vitro* is not prominent *in vivo*.

### Collective Cell Migration of Caudal NPCs *In Vitro*

**Figure 3:**
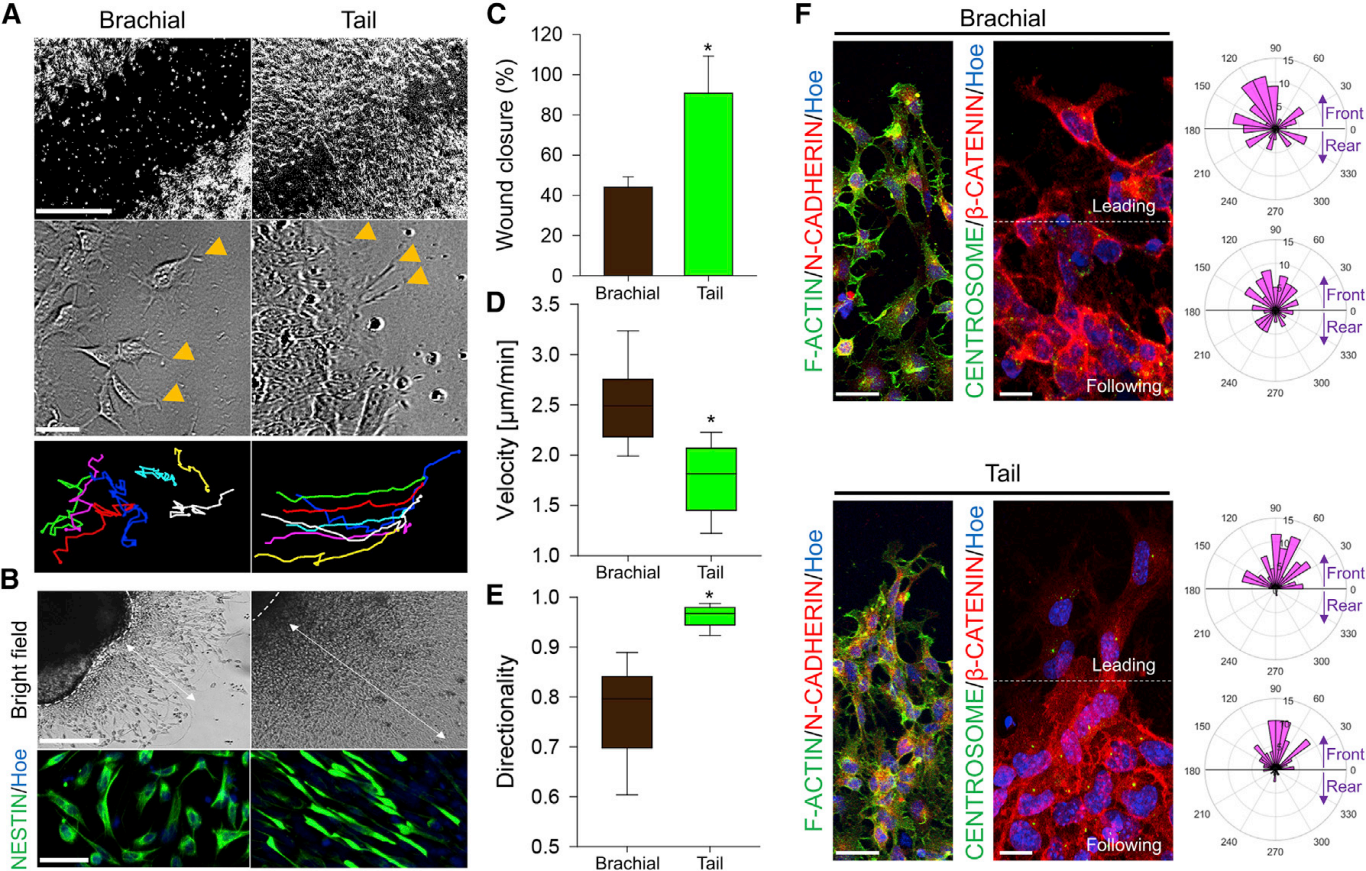
Different Modes of NPC Migration Depending on the AP (A) Images of monolayer brachial (left) or tail (right) NPCs in a scratch assay. Images in the middle are magnified views of scratch borders. Yellow arrowheads indicate the formation of lamellipodia. Line trajectory with different colors represents the trails of individual cells. Scale bars, 200 μm and 70 μm. (B) Explants of brachial and tail NTs were cultured and allowed to migrate for 12 h. Scale bar, 100 μm. The bottom shows NESTIN^+^ (green) migrated NPCs. Nuclei were counterstained with Hoechst 33342 (blue). Scale bar, 20 μm. The dotted lines indicate the margin of the original explant. Double arrows indicate the distance of migration. Number of independent experiments = 3; number of examined explants = 18. (C) Quantification of wound closure rate of NPCs. Raw data were obtained from a series of images at constant time intervals to obtain a percentage. Data are shown as mean ± SD; number of independent experiments = 3; ^∗^p < 0.001 via Student's t test. (D and E) Box plots of the velocity (D) and directionality (E) of cells during the scratch assay. Data are shown as median ± SD; number of independent experiments = 6; ^∗^p < 0.001 via Mann-Whitney rank-sum test. The number of examined cells = 121. (F) Migrating NPCs labeled with nuclei (blue), N-CADHERIN (red), and F-ACTIN (green) or γ-TUBULIN (green, centrosome) and β-CATENIN (red, cell margins). The dotted white lines indicate the border of leading and following cells. Different lengths of the angle bar (polar graphs) represent different grouped cells with a particular angle in the angle histograms. Front (angle 0°–180°) and rear (angle 180°–360°) cells were grouped based on the position of the centrosome. Number of independent experiments = 3; number of examined cells = 311. Scale bar, 20 μm.

We hypothesized that AP-axis-dependent difference in cell adhesion affinity is associated with differential embryonic morphogenetic processes, including NPC migration during trunk elongation. We, therefore, explored the migratory behaviors of NPCs from different levels of the NT (Figure 3). In the scratch assay, the speed of scratch filling is significantly faster in tail-derived NPCs (Figures 3A, 3C and S3A). Quantification of the directionality and the velocity from the single-cell tracking data demonstrated the different strategy of gap filling with distinct modes of cell migration in the two NPC groups. Although the tail-derived NPCs migrate slowly (Figure 3D), they were connected and migrated to the gap together, resulting in faster gap filling (Figure 3E). High-magnification images and time-lapse imaging demonstrated different modes of cell migration in two populations; while migrating brachial-derived NPCs at the leading edge were relatively isolated from their colony and exhibited amoeboid-like random migration, tail-derived NPCs maintained cell-cell adhesion and exhibited collective migration, where cells migrated together in a constant track (Figure 3A, progressive colored lines; and Video S2). Similar results were obtained with explants obtained from different levels of the NT (Figures 3B and S3B) or neurospheres plated on coverslips (Figures S3C–S3F). Collective cell migration is the process by which a group of cells moves in concert, without disrupting their cell-cell interaction and cell polarity. Consistently, we observed that the leading and following tail-derived NPCs were highly polarized and most cells were positioned toward the gap, whereas brachial-derived NPCs showed more randomized polarity (Figure 3F and S2C). On the other hand, both NPC populations expressed similar amounts and distributions of cadherins (Figures S3G, S3H, and S2C), less favoring the idea that cadherin-dependent signaling is responsible for the different cell sorting/migration properties of NPCs.

Video S2. Monitoring the Migration Mode of Individual NPCs Derived from the Tail and Brachial Levels after the Scratch Assay Random and amoeboidal mode of brachial NPC migration is clearly observed in comparison with the collective migration of tail NPCs. Cell migrations were monitored using a Juli stage equipped with a 10× objective lens and placed in a 5% CO2 incubator at 37°C. Images were taken every 10 min for 24 h.

### Dual Gradients Defining the AP Axis Mediate the Cell Adhesion Affinity and Migratory Behavior

To better understand the behavioral differences of NPCs from different NT levels, we aimed to identify the molecular mechanism that regulates the adhesion and migration phenotypes. First, we performed transcriptome comparisons among forebrain-like (N_A_), hindbrain-like (N_H_), and posterior-like (N_P_) NPCs from mouse ESCs using a published dataset (Figure S4A) (Gouti et al., 2014). Remarkably, gene ontology analysis of the N_P_/N_H_ ratio showed significantly enriched gene clusters associated with signaling pathway, including the Wnt/β-catenin pathway (Figure S4B). qRT-PCR analyses of *Wnt*-related genes in brachial- and tail-derived NPCs showed that the expression of genes involved in the Wnt/β-catenin pathway was significantly higher in the tail-derived NPC groups (Figure S4C).

**Figure 4:**
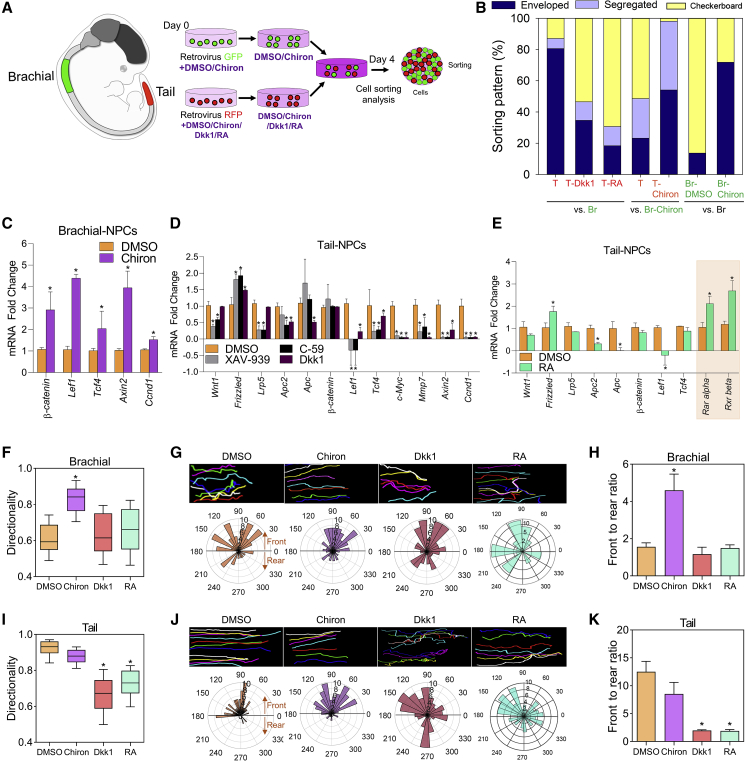
Wnt/β-catenin Signaling Determines AP Gradients of Cell-Cell Adhesions (A) Schematic diagram of E12 embryo, and diagram of cell-sorting assay where cells pre-treated with DMSO, Chiron, Dkk1, or RA were allowed to self-sort based on their adhesion affinity. The gradient of purple color indicates the activation rate of different signals upon chemical treatment. (B) Quantification of the percentage of sorting patterns. NPCs treated with chemicals were from E12 T or Br. Br versus T resulted in significantly (80%) higher enveloped phenotype than segregated 5% and checkerboard 15%. Br versus T-Dkk1 resulted in significantly (58%) higher checkerboard phenotype than segregated 14% and enveloped 28% phenotypes. Br versus T-RA resulted in significantly (70%) higher checkerboard than 12% segregated and 18% enveloped. Br-Chiron versus T resulted in significantly (47%) higher checkerboard phenotype than segregated 30% and segregated 23% phenotypes. Br-Chiron versus T-Chiron E12 resulted in significantly (52%) higher enveloped phenotype than segregated 3% and checkerboard 45%. Br versus Br-DMSO resulted in significantly (88%) higher checkerboard phenotype than enveloped 12% phenotype. Br versus Br-Chiron resulted in significantly (72%) higher enveloped phenotype than checkerboard 28%. Data are shown as percentage; number of independent experiments = 3; number of examined neurospheres = 1,638. (C) RT-PCR analyses present the mRNA fold change in *β-catenin*, *Lef1*, *Tcf4*, *Axin2*, and *Cyclin D1* expression levels upon activation of Wnt signaling with 2 μM Chiron. Data are shown as mean ± SD; number of independent experiments = 3. ^∗^p < 0.001 via Student's t test. (D) qRT-PCR analysis shows the fold change in mRNA levels of *Wnt*-related genes pre-treated with chemicals. Data are shown as mean ± SD; number of independent experiments = 3; ^∗^p < 0.001 via one-way ANOVA. (E) qRT-PCR of *Wnt*-related genes in tail NPCs pre-treated with DMSO or 1 μM RA. Data are shown as mean ± SD; number of independent experiments = 3; ^∗^p < 0.001 via Student's t test. Orange shading indicates the downstream genes of RA signaling. (F and I) Box plots for the directionality of brachial (F) and tail (I) NPCs pre-treated with chemicals during the scratch assay. Data are shown as median ± SD; number of independent experiments = 4; number of examined cells = 401; ^∗^p < 0.001 via one-way ANOVA on ranks. (G and J) Top shows cell trajectory of migrating brachial (G) and tail (J) NPCs, and the bottom shows the angle histograms (polar graph) representing the direction of migrating cells at the leading and following domains. Different lengths of the angle bar represent different grouped cells with a particular angle. Front (angle 0°–180°) and rear (angle 180°–360°) cells were grouped based on the position of the centrosome. Number of independent experiments = 3; number of examined cells = 395. (H and K) Measurement of front-to-rear ratio of brachial (H) and tail (K) NPCs that were determined in polar graphs following chemical treatment. Data are shown as mean ± SD; number of independent experiments = 3; number of examined cells = 395; ^∗^p < 0.001 via one-way ANOVA.

During development, Wnt/β-catenin and RA signaling are well known to instruct the AP axis (Wilson et al., 2009). Both Wnt and RA signals are intermingled, where exposure to RA inhibits the *Wnt* expression *in vivo*. Thus, we tested whether AP-axis-dependent properties of NPCs are regulated by Wnt-RA signaling. The sorting assay revealed a significant increase in checkerboard phenotype by pre-incubation of the tail-derived NPCs with the Wnt inhibitor Dkk1, or RA, indicating that the suppression of Wnt or activation of RA signaling directed caudal NPCs resembling the anterior NPCs (Figures 4A and 4B). Conversely, activation of Wnt/β-catenin signaling with CHIR99021 (Chiron) treatment in brachial-derived NPCs resulted in more checkerboard-like re-aggregates with tail-derived NPCs (Figure 4B). qRT-PCR analyses of *Wnt* (Figures 4C and 4D) and RA-related genes (Figure 4E, orange shaded) confirmed that chemical treatment induced inhibition or activation altered their gene expression profiles accordingly. Notably, different Wnt inhibitors suppressed *Wnt* downstream genes to different extents, presumably depending on their targets in the Wnt/β-catenin signaling cascade, but they all led to the strong inhibition of the far-downstream effector genes (such as *c-Myc*, *Mmp7*, *Axin2*, and *Ccnd1*). Interestingly, RA treatment also suppressed the downstream genes of Wnt signaling, while it did not affect the expression of *Wnt* receptors (Figure 4E). However, activation of Wnt/β-catenin signaling did not modify their endogenous *Hox* code (Figure S4D), indicating that the changes in cell adhesion affinity due to Wnt signaling are not associated with the altered regional specification.

Next, we asked whether the differences in cell adhesion affinity among adjacent NPCs in the lumbar domain are also controlled by Wnt signaling. To address this, we isolated lumbar-derived NPCs from TCreERT2:Rosa-EGFP lineage tracing mice and explored the dynamic interaction between GFP^+^ and GFP^−^ NPCs upon Chiron treatment (Figure S5A). Interestingly, the checkerboard phenotype was significantly increased to 70% in the Chiron-treated group compared with the DMSO-treated group (Figure S5B), indicating that early-born GFP^−^ NPCs are still sensitive to Wnt signaling, and Wnt activation can override the birthdate-dependent difference in cell adhesion affinity between GFP^−^ and GFP^+^ NPCs within the lumber spinal cord. Therefore, it appears that NPCs progressively lose their sensitivity to Wnt, which possibly contributes to the ordered arrangement of NPCs in regions of similar Wnt concentration.

We next analyzed whether the modes of NPC migration are also altered by modulation of Wnt-RA signaling (Figures 4F–4K). Time-lapse imaging of migrating brachial-derived NPCs treated with Chiron showed the conversion of their mode from amoeboidal to collective migration, as evidenced by enhanced directionality and cell polarization (Figures 4F–4H, S5D, S5E and Video S3). In contrast, the suppression of Wnt signaling by Dkk1, XAV-939, or RA did not affect the migration mode, indicating that brachial-derived NPCs do not have endogenous Wnt activation, but their migration mode can be caudalized by Wnt activation. Conversely, upon treatment with Dkk1, XAV-939, or RA, tail-derived NPCs failed to maintain collective migration, with a significant reduction in directionality and cell polarization, while Chiron treatment did not significantly affect their migration mode (Figures 4I–4K, S4F, S4G, S5D, and S5E). Other activators of caudal signaling, including FGF8b and GDF11, did not affect the migration of NPCs (Figure S4E). Taken together, these data demonstrate that the Wnt-RA gradient instructs the differential cell adhesion/migration properties of NPCs in the elongating NT and may instruct the morphogenesis of the NT via alterations in NPC behavior, such as cell adhesion and migration.

Video S3. Monitoring the Migration Mode of Individual NPCs Derived from the Brachial Level Pre-treated with Chiron for 4 Days before the Scratch Assay Adhesion between NPCs is enhanced upon Wnt activation with Chiron as evidenced by a collective-like migration phenotype. Cell migrations were monitored using a Juli stage equipped with a 10× objective lens and placed in a 5% CO2 incubator at 37°C. Images were taken every 10 min for 24 h.

### Differential Expression of ECM Genes in the NPC Populations

**Figure 5:**
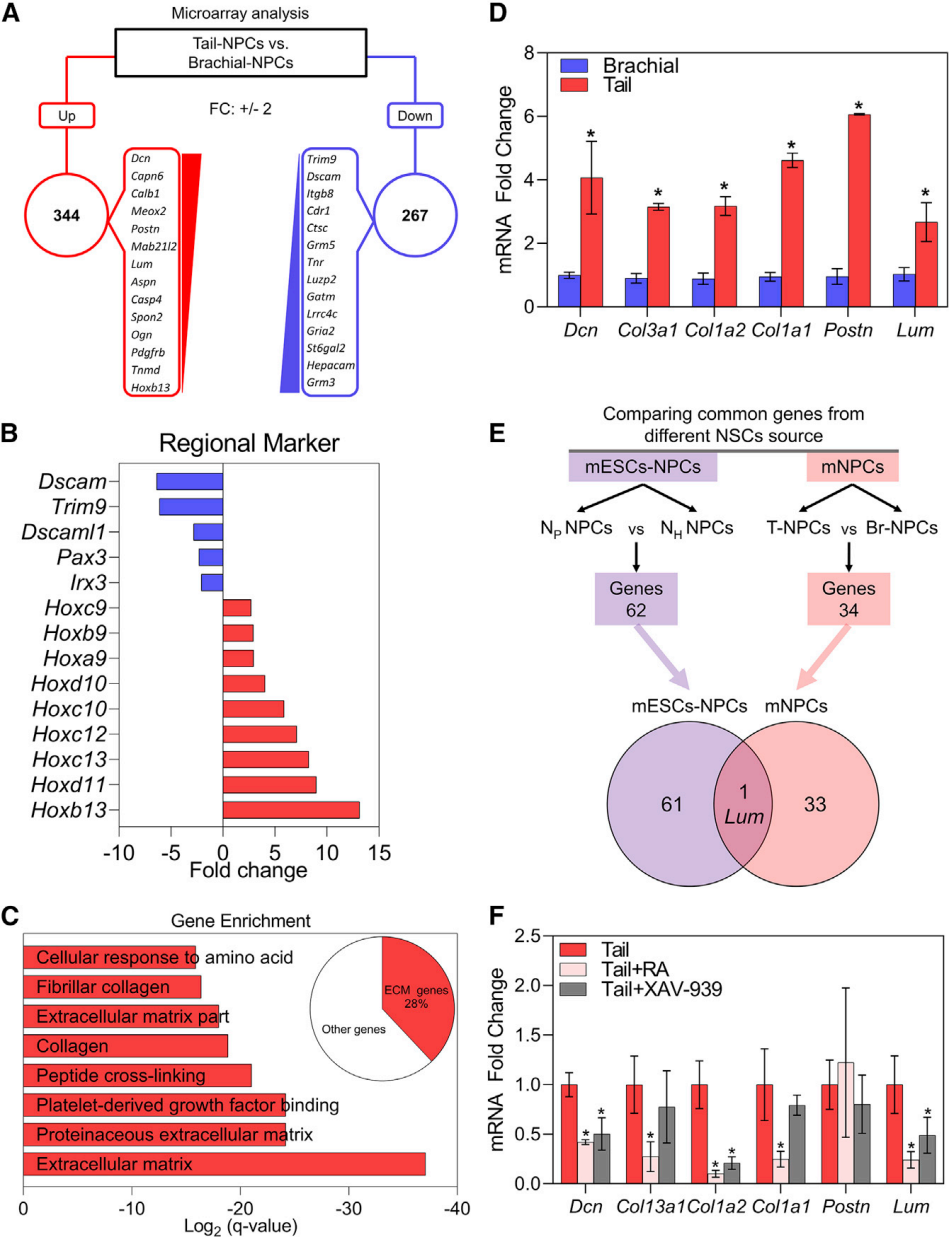
Transcriptome Analysis of Mouse NPCs Uncovered ECM Enrichment (A) Diagram of results of NPC microarray analysis. Top up- and downregulated genes (red and blue boxes, respectively) are listed. Up- and downregulated genes were selected based on the fold change of ≥2 and ≤ −2, respectively. (B) Fold-change level of various markers of rostral and caudal domains of the embryos obtained from microarray data to validate regional identity. (C) Bar graphs presenting the enriched gene ontology terms of upregulated genes in tail NPCs compared with brachial NPCs. Pie chart shows the percentage of ECM genes. (D) Relative mRNA levels of ECM genes identified in (C). Data are shown as mean ± SD; number of independent experiments = 3; ^∗^p < 0.05 via Student's t test. (E) Venn diagram showing the overlap of upregulated genes in mouse embryonic NPCs (red) *in vivo* and NPCs derived from ESCs *in vitro* (purple). (F) Relative mRNA levels of ECM genes in tail NPCs pre-treated with chemicals. Data are shown as mean ± SE; number of independent experiments = 3; ^∗^p < 0.05 via one-way ANOVA on ranks.

**Figure 6:**
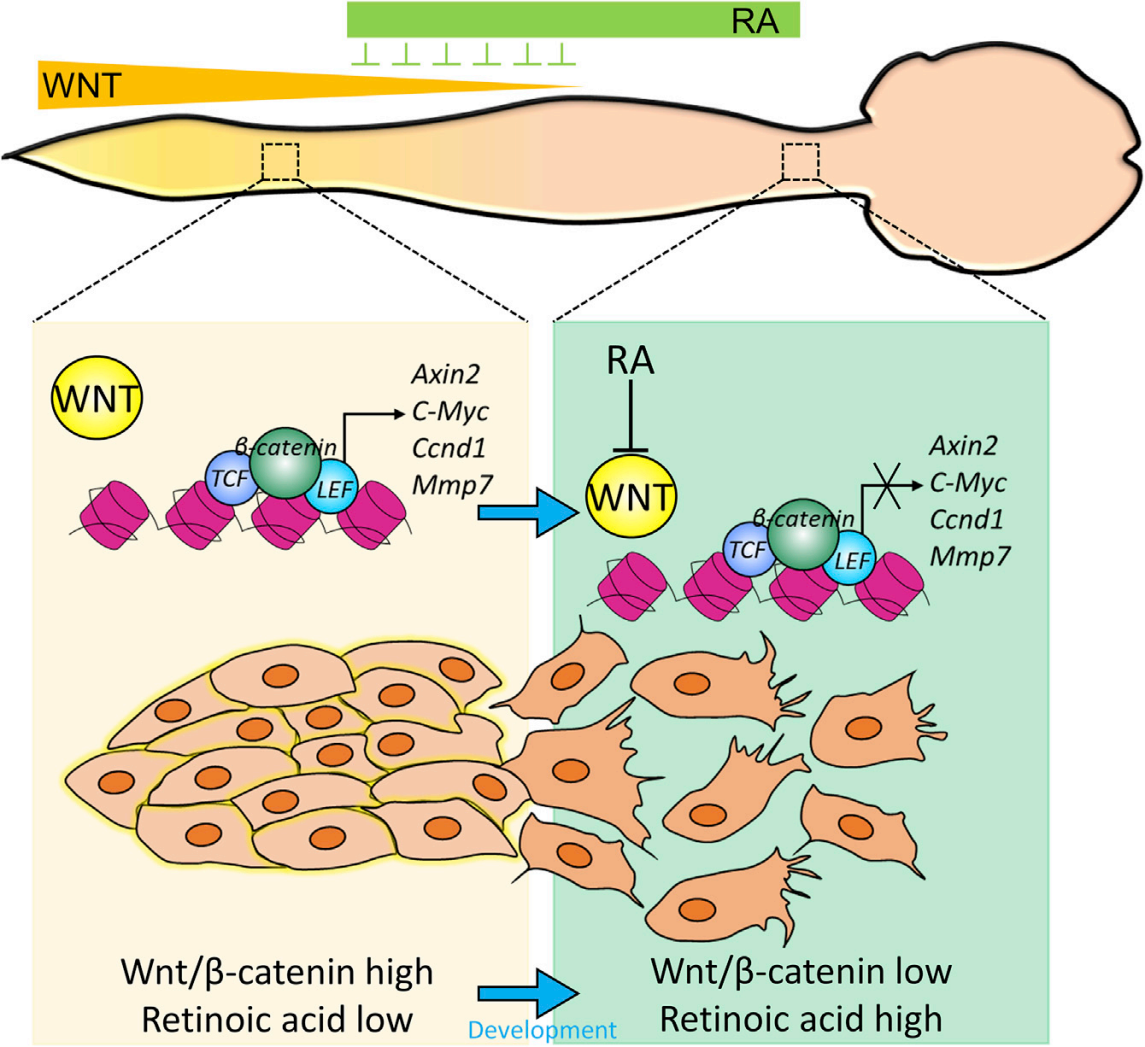
Schematic Summary of Wnt-RA Network for the Regulation of AP Gradient of NPC Adhesion Properties In the developing spinal cord, the newly produced NPCs from NMPs are exposed to high Wnt signaling to promote cell-cell adhesion by allowing collective anterior movement. By continual generation of NPC clusters, early-born NPC clusters move away from Wnt signaling and are eventually exposed to RA, which antagonizes the Wnt signal, causing a progressive loss of Wnt-signaling-responsive genes. Thus, NPCs start losing responsiveness to Wnt, which sharpens the cell adhesion gradient.

To uncover the mechanisms that mediate the strong cell interactions among caudal NPCs, we compared the transcriptomes of NPCs derived from brachial and tail (Figure 5A). We found 344 genes enriched in the tail-derived NPCs and 267 genes enriched in the brachial-derived NPCs (Table S1). Regional markers from microarray data validated the rostrocaudal identity of the NPCs (Figure 5B). We isolated tail-NPC enriched genes and clustered them according to their functional annotations. Gene enrichment analysis revealed that many extracellular matrix (ECM)-related genes were enriched in the tail-NPC group (Figure 5C), with 28% of ECM genes among the top 10% of upregulated genes (Figure 5C, pie chart), whereas brachial NPCs were enriched with neuronal regulation-related genes (Figure S6). Significantly strong expression of the ECM genes in the tail-derived NPCs was further validated by qRT-PCR (Figure 5D). In addition, we compared our own dataset with the RNA-sequencing dataset obtained from the comparison of N_H_ and N_P_ (Table S2) (Figure 5E). We identified one overlapped gene of the two groups, which is an ECM gene, *lumican* (*Lum*), further confirming the enrichment of ECM in caudal NPCs. Since the RA and Wnt inhibitors treatment reduced the adhesive properties of tail NPCs, we also examined whether RA and XAV-939 affect the expression of ECM genes in tail NPCs. qRT-PCR of tail NPCs treated with RA/XAV-939 daily for 4 days showed a significant reduction in ECM genes compared with untreated tail NPCs (Figure 5F). Collectively, these results revealed the enrichment of ECM in tail NPCs, which is under the control of Wnt/RA signaling, which strengthens the possibility that ECM is a critical factor in the cell adhesion and collective migration of caudal NPCs (Figure 6).

## Discussion

In this study, we identified the gradual alteration of cell adhesion and migration properties of NPCs according to the AP axis, which is controlled by the Wnt-RA gradient (Figure 6). This spatiotemporal gradient is important for axial elongation, providing a model of how NT elongation and regional specification is coordinated by the axis-determining Wnt-RA signals. Interestingly, these differences in cell adhesion affinity between rostral and caudal NPCs are maintained in human pluripotent stem cell-derived NPCs *in vitro*, indicating the importance of these NPC adhesion properties in human spinal cord development. Considering that cell-cell adhesion is one of the fundamental events for histogenesis, our current findings open new insights for understanding the cellular mechanism of NT elongation and AP-axis-dependent organization of spinal cord morphogenesis.

During organogenesis and trunk elongation, the NT (which forms the CNS) undergoes both growth and axial elongation. The AP axis domains are generated from two major sources of NPCs, the rostral NECs and caudal NMPs. However, we do not believe that different cell-cell adhesion affinity is directly associated with NPC origin. For instance, most NPCs in the caudal (lumbar) level of the spinal cord are generated from NMPs; however, they show differences in cell adhesion properties depending on their birthdate. Thus, we favor the idea that this difference is primarily controlled by local morphogenetic cues as discussed below. The difference in cellular adhesion is a driving factor for cells to undergo morphogenesis throughout development. Embryonic tissues are made of cells derived from one or multiple lineages, and physical separation among these cells is an essential driver for a proper embryonic development (Fagotto, 2014). The DITH explains that differences in contractility of the cortical actomyosin cytoskeleton proteins or adhesion are the parameter that determines tissue separation (Brodland, 2002). This study characterized the differential cortical adhesion affinities among spinal cord tissue and uncovered the enrichment of ECM in caudal NPCs having strong cell-cell interactions, which is in consistent with the DITH model. NPCs with a difference in cellular adhesion intercalate during convergence extension to establish the rostral neural folding, and many adhesion genes have been identified to cause rostral NT defects upon mutation (Copp et al., 2010). On the other hand, the process of secondary neurulation mediates the secondary phase of spinal cord development, where NPCs have the ability to aggregate and fuse to the caudal end of the rostral NT during NT elongation. The differential adhesion properties of these cells are proposed to be one of many driving factors involved, such as signaling pathways and morphogen secretion (Colas and Schoenwolf, 2001). Our study is the first to provide the evidence of caudal NPCs with strong adhesion affinity during secondary neurulation phase and uncover Wnt-RA as a key driving pathway to mediate cell-cell contacts.

Cell adhesion affinity affects many aspects of cellular behavior, including cell migration. In general, organogenesis involves morphogenetic movements, in which a cluster of cells migrate in a coordinated manner to form a particular organ (Weijer, 2009). Collective migration has a key role during morphogenesis, where a group of cells migrates together in the same direction at similar speeds. Hence, cell-cell adhesion among cell groups coordinates integrity and motility (Vicente-Manzanares et al., 2009). Although NPC movement *in vivo* in mouse or other vertebrate embryos has not yet been described, the organization for trunk elongation by NMP-derived mesoderm has been extensively addressed (Oginuma et al., 2017). Paraxial mesodermal progenitors (PMs), which are also derived from NMPs, do not exhibit collective migration but exhibit extracellular matrix-dependent disorganized cell motion, which promotes the symmetrical division of progenitors to meet the demand of somitogenesis. This disorganized motion of PMs is mediated in part by a sharp reduction in CADHERIN 2 on the cell surface, a protein known to increase cell adhesion affinity (Das et al., 2017). As PMs join the posterior domain of a newly forming somite, cell motion decreases, representing a transition from a viscoelastic fluid to a viscoelastic solid, which is responsible for body elongation (McMillen and Holley, 2015). Our study provides evidence that caudal NPCs exhibit stronger adhesion affinity than rostral NPCs, hence, caudal NPCs exhibit a collective migration manner. Consistent with our data, a recent study with amniotes reported that NPCs moved at similar velocities with collective tectonic movement during NT elongation *in vivo* (Bénazéraf et al., 2017), suggesting that NPCs migrate together rostrally in a collective manner to build up the elongating NT. Therefore, collective migration appears to be a unique feature of early NPCs that exhibit epithelial-like cell adhesion and polarization (Sidhaye and Norden, 2017).

Local signaling for NPC production plays essential roles in NT formation, neural axis elongation, and, later, AP axis patterning. A recent study highlighted the importance of caudal-to-rostral cell migration as essential to promote axial elongation (Denans et al., 2015). An extensive body of information supports the importance of Wnt signaling in the axial elongation. *In vitro* and *in vivo*, high Wnt signaling enhances the pool of caudal NPC formation from the NMPs (Garriock et al., 2015). Ablation of *Bra-T* in NMPs impairs cell migration out of the tail bud and compromises axial elongation, which is mediated by Wnt signaling (Martin and Kimelman, 2008). Wnt signaling also plays a pivotal role in cell migration during gastrulation (Schambony and Wedlich, 2003). For instance, the convergent extension movement of a neurulating embryo involves narrowing and lengthening of cell populations, and blocking of Wnt signaling alone results in the inhibition of convergent extension movements (Kühl et al., 2001). Our experiments demonstrate a major impact of Wnt signaling on the adhesion of NPCs, suggesting that Wnt controls the mode of NPC migration during neural axis elongation. Interestingly, adjunct PM motility is mediated by FGF signaling (Oginuma et al., 2017), which indicates that two major progeny lineages of NMPs, NPCs, and PMs, uniquely respond to different local signaling within similar domains. Elongation of the NT gradually positions the NPCs to a high concentration zone of another signal, the RA, which is released from paraxial mesoderm-derived somites (Olivera-Martinez et al., 2012). Subsequently, NMP maintenance is repressed when RA-producing segmented somites are in close vicinity of the tail bud, marking the termination of axial elongation (Das et al., 2017; Denans et al., 2015). Thus, the exposure of the caudal tail bud to an increased concentration of RA inhibits Wnt expression, resulting in the depletion of NMPs and the arrest of axis elongation *in vivo* (Olivera-Martinez et al., 2012). Consistent with this model, tail-derived NPCs respond to exogenous treatment of RA, alter the endogenous expression of Wnt signaling-related genes, attenuate the adhesion affinity, and convert cell migratory modes from collective tectonic to amoeboidal. This indicates that adhesion and migratory properties of caudal NPCs are tightly regulated by the local environmental gradient of Wnt and RA. We also observed that NPCs from a similar level of the NT exhibit different adhesion properties depending on their timing of production from the NMPs. During the axial elongation, newly produced NPCs from NMPs progressively move to the rostral domain where they are exposed to higher RA and lower Wnt. It is known that RA inhibits Wnt signaling at the β-catenin level, where RA increases the expression of *RARα* and *RXRβ*, which compete with TCF/LEF binding for β-CATENIN, and as a result retinoids decrease the activation of the *LEF*/*TCF* family of transcription factors (Lu et al., 2009). This activity of RA is independent of the APC tumor suppressor and ubiquitination-dependent degradation of cytoplasmic β-CATENIN (Easwaran et al., 1999). In our analyses, we found that RA altered these gene expression levels, suggesting that RA may antagonize Wnt signaling via induction of *Rar* expression. Since RA antagonizes Wnt activation, older NPCs that are exposed to RA longer may downregulate the genes required for response to Wnt signaling and become less sensitive to Wnt signaling than younger NPCs. In agreement with this model, cell adhesion property was reduced in older NPCs within the lumbar domain. Such cellular gradients may assist in aligning NPCs along the AP axis depending on their cell adhesion property during axial elongation.

It is known that Wnt contributes to proper anterior-posterior patterning during trunk elongation (Mulligan and Cheyette, 2012), and high Wnt signaling positively supports the pool of neural and mesodermal progenitors (Garriock et al., 2015). For instance, Tet-deficient embryos exhibit hyperactivation of Wnt signaling, leading to aberrant differentiation of NMPs into mesodermal lineage at the expense of neural lineage (Li et al., 2016). Another study demonstrated that *Wnt3a* positively supports the progenitor state of both mesodermal and neural lineages *in vivo* (Garriock et al., 2015). This commitment is associated with the transient rise in Wnt/β-catenin signaling (Turner et al., 2014). Therefore, it remains to be illustrated whether different activation levels of Wnt/β-catenin signaling or different subtypes of *Wnts* determine the regulation of mesoderm at the expense of neural tissue.

Using transcriptome analyses of two datasets from the comparisons of (1) NPCs derived from different levels of embryonic NT and (2) brachial- and lumbar-like NPCs derived from mouse ESCs, we identified that ECM molecules may mediate the adhesion gradient of NPCs in the AP axis. This is consistent with a previous report showing that ECM drives cell aggregate formation *in vitro* (Caicedo-Carvajal et al., 2010). The common analysis with previous RNA-sequencing data of rostral and caudal NPCs derived from mouse ESCs identified *Lum* as a common gene. LUM belongs to the SLRP family of proteins that contribute to the ECM complex (Chen and Birk, 2013), and it is reported to be localized in the connective tissues as a component of the ECM and also to play regulatory roles in collagen fibrillogenesis, the chemokine gradient, wound healing, and epithelium-mesenchyme transition under physiological and pathophysiological conditions (Yamanaka et al., 2013). In parallel, a posterior-to-anterior gradient of extracellular pH measurement during trunk axial elongation has been recently reported (Oginuma et al., 2017), suggesting a gradient of ECM protein expression along the AP axis. In the same context, LUM has been shown to positively regulate the gradient of cell adhesion (D’Onofrio et al., 2008).

In summary, the current study uncovered a novel link of Wnt-RA signaling and NPC adhesion and collective migration, which is the cellular basis of axial elongation of NT. Our present study provides new insight into the importance of ECM molecules in cellular behavior along with lineage determination mediated by local signaling, Wnt, and RA for axial NT elongation during embryonic development.

## Experimental Procedures

Detailed experimental procedures are provided in the Supplemental Information.

### Mice and Embryos

For transgenic mice, the TCreERT2 transgenic mouse line used in this work was generated as described previously (Anderson et al., 2013). The TCreERT2 transgenic and Rosa-EGFP reporter mice were purchased from Jackson ImmunoResearch Laboratory. Pregnant females were subjected to treatment with 0.175 mg/g TAM. Pregnant females were then sacrificed at E12 to dissect out the embryos and processed for *in vitro* and *in vivo* experiments.

### Embryonic Neural Progenitor Culture

Embryonic NPC culture was performed as described previously (Shaker et al., 2015). Dissected tissues were then incubated for 15 min with Accutase at 37°C to generate single cells, which were seeded and expanded into neurospheres on ultra-low-attachment culture dishes. For viral infection, the virus concentration was adjusted to 1 × 10^9^ transducing units/mL and GFP or RFP was injected into NPCs at day 1 as described previously (Kim et al., 2015). Labeled neurospheres were dissociated and single cells were used for the sorting assay.

### Cell Sorting Assay

Single cells were co-cultured at a 1:1 ratio with final plating density of 5 × 10^5^ cells/2 mL N2 medium supplemented with epidermal growth factor/basic fibroblast growth factor. De-mixed cells were co-cultured for 4 days before quantification of sorting phenotypes. For pre-treatment, NPCs at passage 0 were pre-treated with DMSO, 3 μM Chiron, 200 ng/mL DKK-1, 1 μM XAC-939, 1 μM Wnt-C59, or 1 μM RA for 4 consecutive days. Pre-treated NPCs were then passaged and re-aggregated at passage 1 for 4 days to promote the formation of sorting phenotypes.

### Statistical Analysis

Data are expressed as the mean ± SD of the mean of independent experiments. Statistical analysis was performed using Sigma Plot 12.5 software. Minimal statistical significance was defined at p < 0.05.

### Data and Code Availability

The accession number for the microarray reported in this paper is GEO: GSE132089.

## Author Contributions

M.R.S. designed and performed the experiments, analyzed the data, and wrote the manuscript. J.H.L., S.H.P., J.Y.K., G.H.S., and J.W.S. performed additional experiments. B.H.P., I.J.R., and H.K. provided critical experimental materials and revised the manuscript. W.S. supervised M.R.S., designed the experiments, and wrote the paper.

## Supplemental Information

Document S1. Supplemental Experimental Procedures, Figures S1–S6, and Table S3

Table S1. List of Up- and Downregulated Genes in Tail and Brachial NPCs, Related to Figure 5 fc, fold change.

Table S2. List of Up- and Downregulated Genes in N_P_ and N_H_ NPCs, Related to Figure 5 fc, fold change.

Document S2. Article plus Supplemental Information

## Footnotes

Supplemental Information can be found online at https://doi.org/10.1016/j.stemcr.2020.08.016.
