# Supplementary material for: Anteroposterior Wnt-RA Gradient Defines Adhesion and Migration Properties of Neural Progenitors in Developing Spinal Cord
Source: Stem Cell Reports. 2020 Sep 24;15(4):898–911. doi: 10.1016/j.stemcr.2020.08.016 (PMC7562945; doi:10.1016/j.stemcr.2020.08.016)
Supplement: Document S1. Supplemental Experimental Procedures, Figures S1–S6, and Table S3 [file mmc1.pdf]

Stem Cell Reports, Volume *15*

Supplemental Information

Anteroposterior Wnt-RA Gradient Defines Adhesion and Migration Properties of Neural Progenitors in Developing Spinal Cord

Mohammed R. Shaker, Ju-Hyun Lee, Si-Hyung Park, Joo Yeon Kim, Gi Hoon Son, Jong Wan Son, Bae Ho Park, Im Joo Rhyu, Hyun Kim, and Woong Sun

SUPPLEMENTAL INFORMATION

Anteroposterior Wnt-RA Gradient define adhesion and migration properties of neural progenitors in developing spinal cord

Mohammed R.Shaker, Ju-Hyun Lee, Si-Hyung Park, Joo Yeon Kim, Gi Hoon Son, Jong Wan Son[5], Bae Ho Park, Im Joo Rhyu, Hyun Kim, and Woong Sun

Supplementary Figures

Figure S1

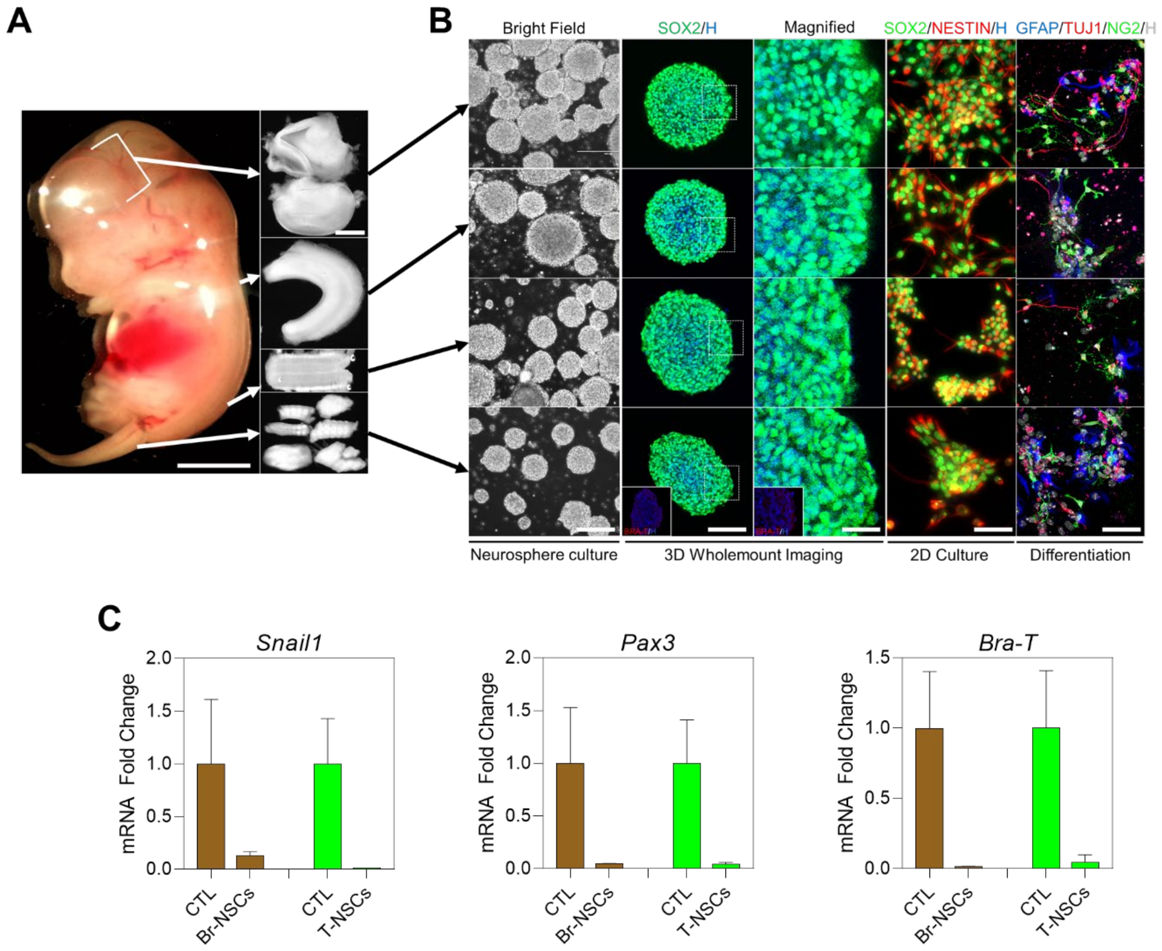

Figure S1. Isolation and Verification of Embryonic NPCs from Different AP Levels. (Related to Figure 1)

(A) E12 mouse embryo that was used to dissect the neural tissues (Right white color tissues) to use as initial materials for neurospheres culture. White arrows indicate the level of CNS domain along the AP axis. Scale bar is 1 mm, magnified images scale bar is 100 µm.

(B) Representative images of neurospheres that were expanded from cortex, brachial, lumbar and tail neural tissues. Stemness and multipotency of NPCs from brain, lumbar and tail levels was confirmed either by the expression of stemness markers SOX2 (Green) using wholemount imaging, or SOX2 (Green) and NESTIN (Red) using immunocytochemistry staining, or by the generation of various neural cells such as astrocytes, (GFAP, Blue), neurons (TUJ1, Red), and oligodendroblasts (NG2, Green) and counterstained with Hoechst 33342 (Blue) upon removal of growth factors, respectively. Scale bar is 15 µm. Magnified images in tail-derived neurospheres as insets shows the absence of BRA-T (Red) immunoreactivity. Nuclei were counterstained with Hoechst 33342.

(C) Relative mRNA level of neural crest genes (*Snail* and *Pax3*) and *Bra-T* gene were analyzed using qPCR in brachial and tail-derived NPCs compared to their neural tissues domains as a control. Data are shown as mean ± s.d; Number of independent experiments =3.

Figure S2

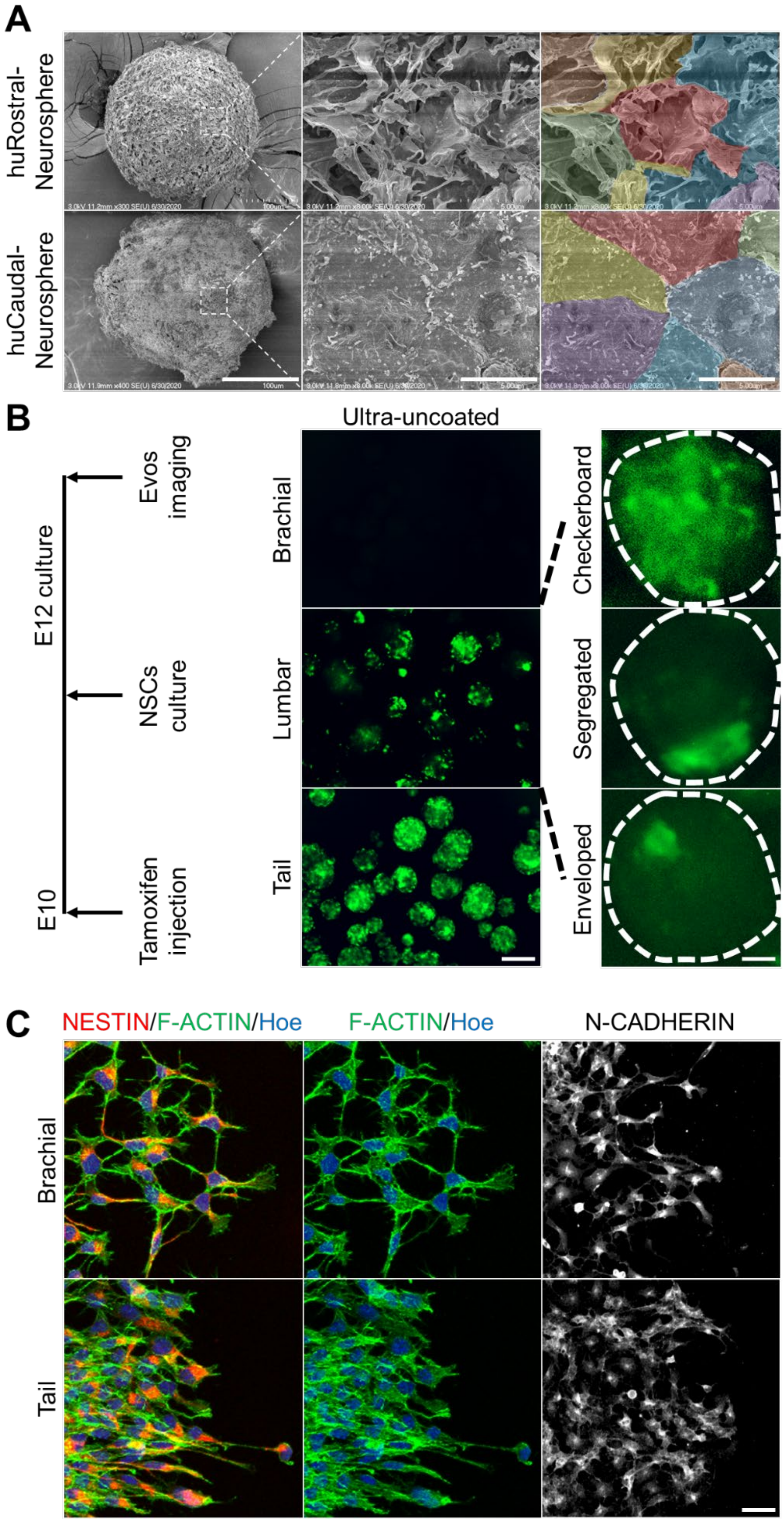

A
huRostral-Neurosphere
huCaudal-Neurosphere
B
E12 culture
E10
Evos imaging
NSCs culture
Tamoxifen injection
Ultra-uncoated
Brachial
Lumbar
Tail
Checkerboard
Segregated
Enveloped
C
NESTIN/F-ACTIN/Hoe
F-ACTIN/Hoe
N-CADHERIN
Brachial
Tail

Figure S2. Culturing Rostral and Caudal NPCs of TCreERT2:Rosa-EGFP embryos. (Related to Figure 1 and Figure 2 and Figure 3)

(A) Scanning electron microscope images of human rostral and caudal neurospheres. Left images show entire neurospheres and the magnified images of dotted squares are shown in the middle. Images with the pseudo coloring of each cell are on the right. Left image scale bar is 100 µm. Magnified image scale bar is 5 µm. The number of examined neurospheres = 9.

(B) Neurospheres from TCreERT2:Rosa-EGFP embryos (E12) injected with tamoxifen at E10. Sorting properties of lumbar- NPCs born at E10 vs. lumbar- NPCs born at the earlier embryonic time. White dotted lines show the margin of neurospheres. Black dotted lines show the selection of different neurospheres from the lumbar group. Scale bar is 200µm. Scale bar is 50µm for magnified images of selected neurospheres. Number of examined neurospheres = 735.

(C) Representative images of migrating NPCs labeled with F-ACTIN (Green), NESTIN (Red), N-CADHERIN (Grey) and counterstained with Hoechst 33342 (Blue) at 1h of scratch migration. Scale bar is 20 µm.

Figure S3

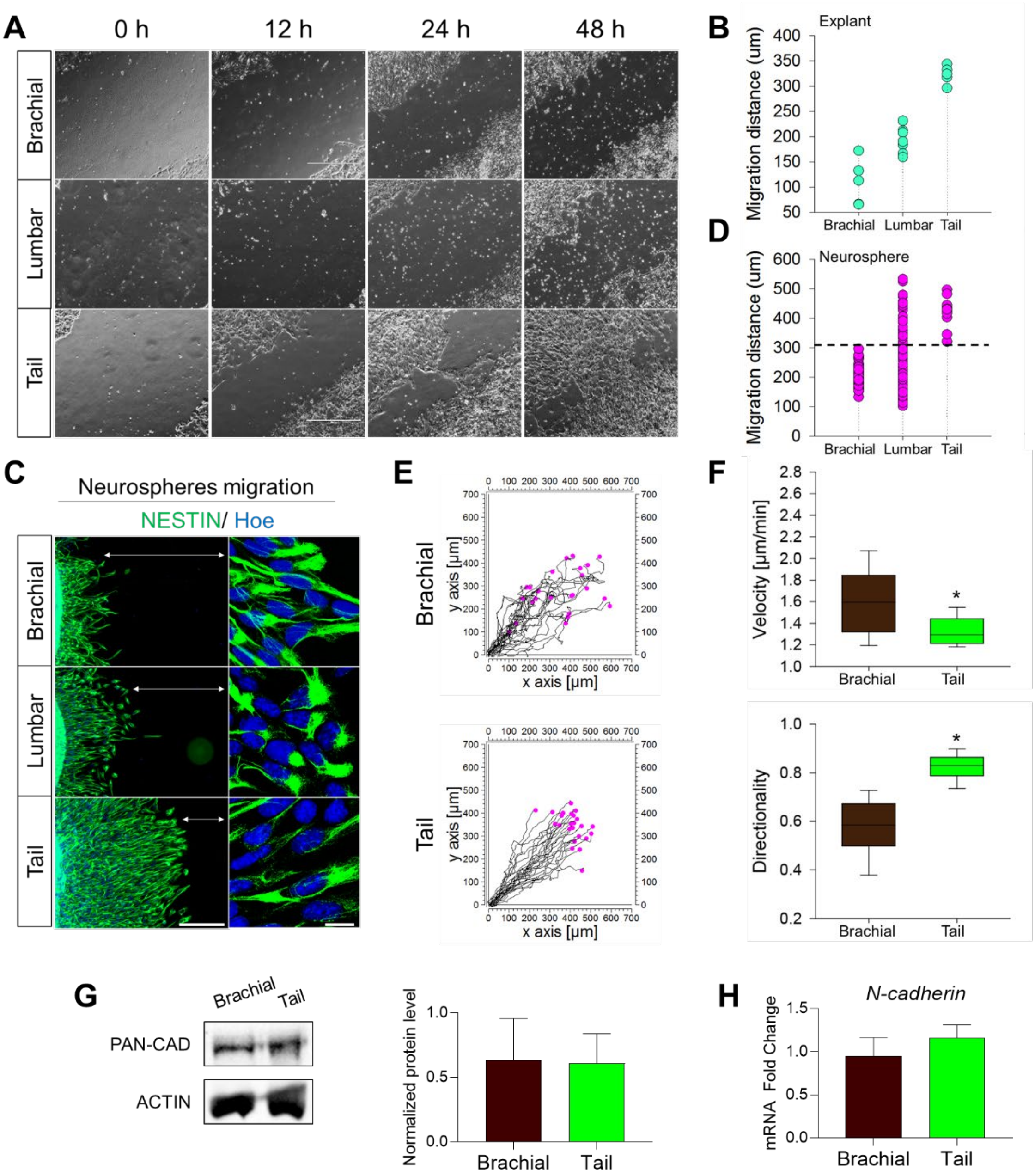

Figure S3. Different Assays of Embryonic NPC Migration. (Related to Figure 3)

(A) Representative images from different time points during the scratch assay. Scale bar is 200 µm.

(B) Dot plots of migration distance of cells from explants in Figure 3E. Individual dots represent measurements from single explant. Number of independent experiments = 3; Number of examined explants = 18.

(C) Representative images of migrating NPCs labeled with NESTIN (Green) and counterstained with Hoechst 33342 (Blue) at 6h of migration. Magnified images of the edge of neurospheres show the polarized morphology of migrating NPCs. Double arrows indicate the distance of migration length across groups. Scale bar is 30 µm, and the magnified scale bar is 3 µm.

(D) Dot plots of migration distance of cells from the neurospheres. An individual dot represents the measurement of a single neurosphere. Number of independent experiments = 3; Number of examined neurospheres = 150.

(E) Live imaging of individual cell behavior from the neurosphere. Individual pink dots represent the behavior of individual cells out of a single neurosphere. Number of examined cells = 52.

(F) Box plots of the measurements of velocity and directionality of NPCs using neurospheres migration assay to show the migration rate and behavioral differences between two groups. Data are shown as the median ± s.d, Number of independent experiments = 3, Number of examined cells = 52, *P < 0.001 via Mann-Whitney Rank Sum Test.

(G) Western blots of PAN-CADHERIN in NPCs. Actin was used for normalization. The right bar graph shows the quantification of Pan-cadherin level. Data are shown as mean ± s.d. The number of independent experiments=3.

(H) Relative mRNA level of *N-cadherin* analyzed using qPCR in brachial and tail-derived NPCs. Data are shown as mean ± s.d; Number of independent experiments=3.

Figure S4

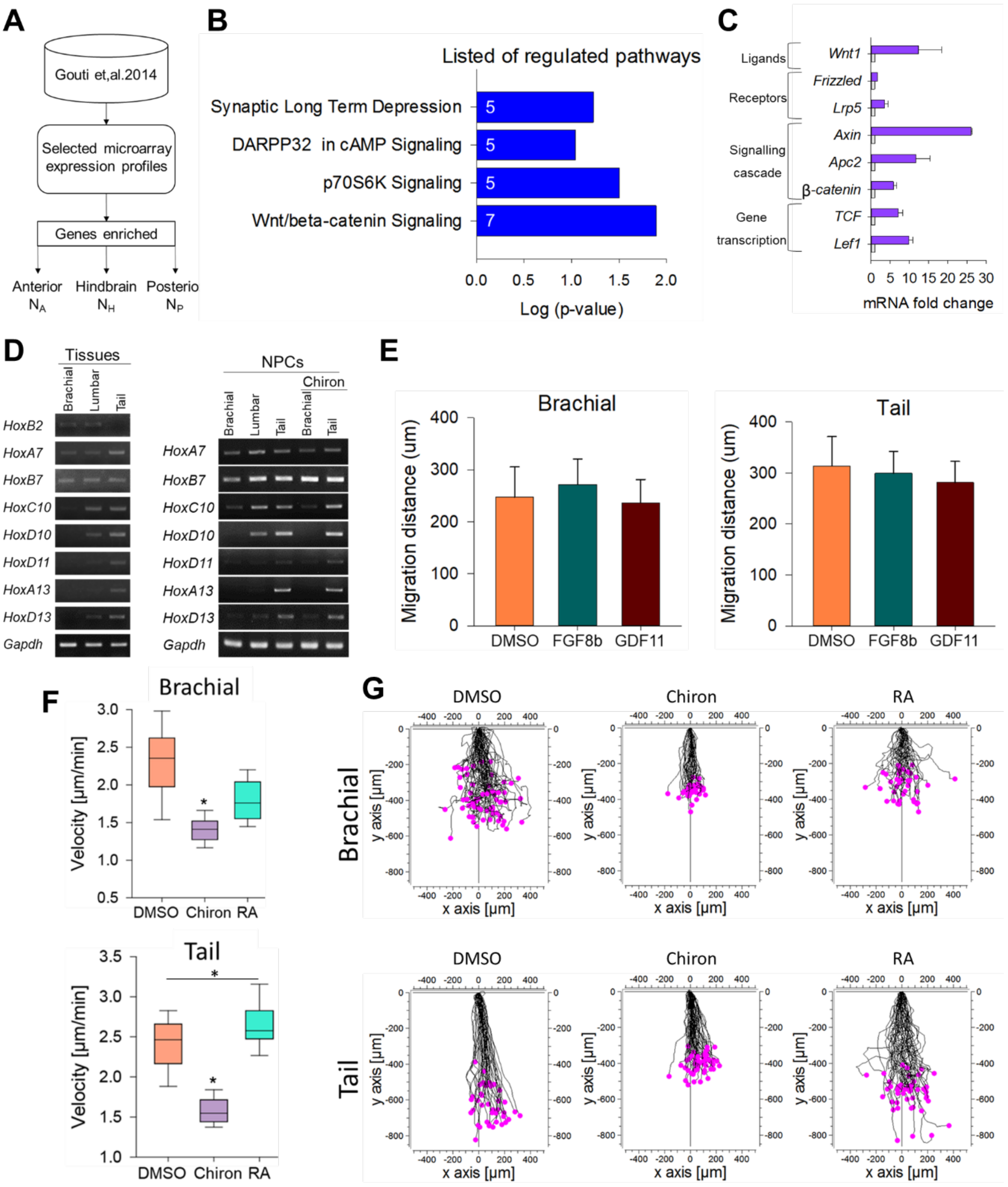

Figure S4. Responsiveness of Embryonic NPCs to Exogenous Activation and Inhibition of Wnt and RA Signalings. (Related to Figure 4)

(A) Flow chart shows the method of RNA-seq selection from published raw data (Gouti et al., 2014).

(B) List of signaling pathways substantially enhanced in caudal NPCs detected from the NP/NH expression profile. Numbers in each bar indicate the number of differentially expressed genes in each category.

(C) qRT-PCR of *Wnt*-related genes. Data are shown as mean ± s.d. from 3 independent experiments.

(D) RT-PCR analyses of *Hox* genes expression in tissues and cultured NPCs. Results indicate the activation of Wnt signaling in brachial NPCs with Chiron did not alter their AP regional identity.

(E) Measurements of migration distance of NPCs generated from brachial and tail upon exogenous treatments of 200 ng of FGF8b or 50 ng GDF11. Data are shown as mean ± s.d.; Number of independent experiments = 3; Number of examined neurospheres = 20.

(F) Box plots show the calculation of the velocity of NPCs pretreated with either DMSO, Chiron or 1μM RA before the scratch assay. Single measurement was obtained from the single migrating cell. Data are shown as median ± s.d.; Number of independent experiments = 3; *$P < 0.001$ via One Way ANOVA on ranks.

(G) Live imaging of individual cell behavior from the scratch assay upon chemical treatments to show the persistence and migration length of individual brachial and tail-derived NPCs. Number of examined cells = 325.

Figure S5

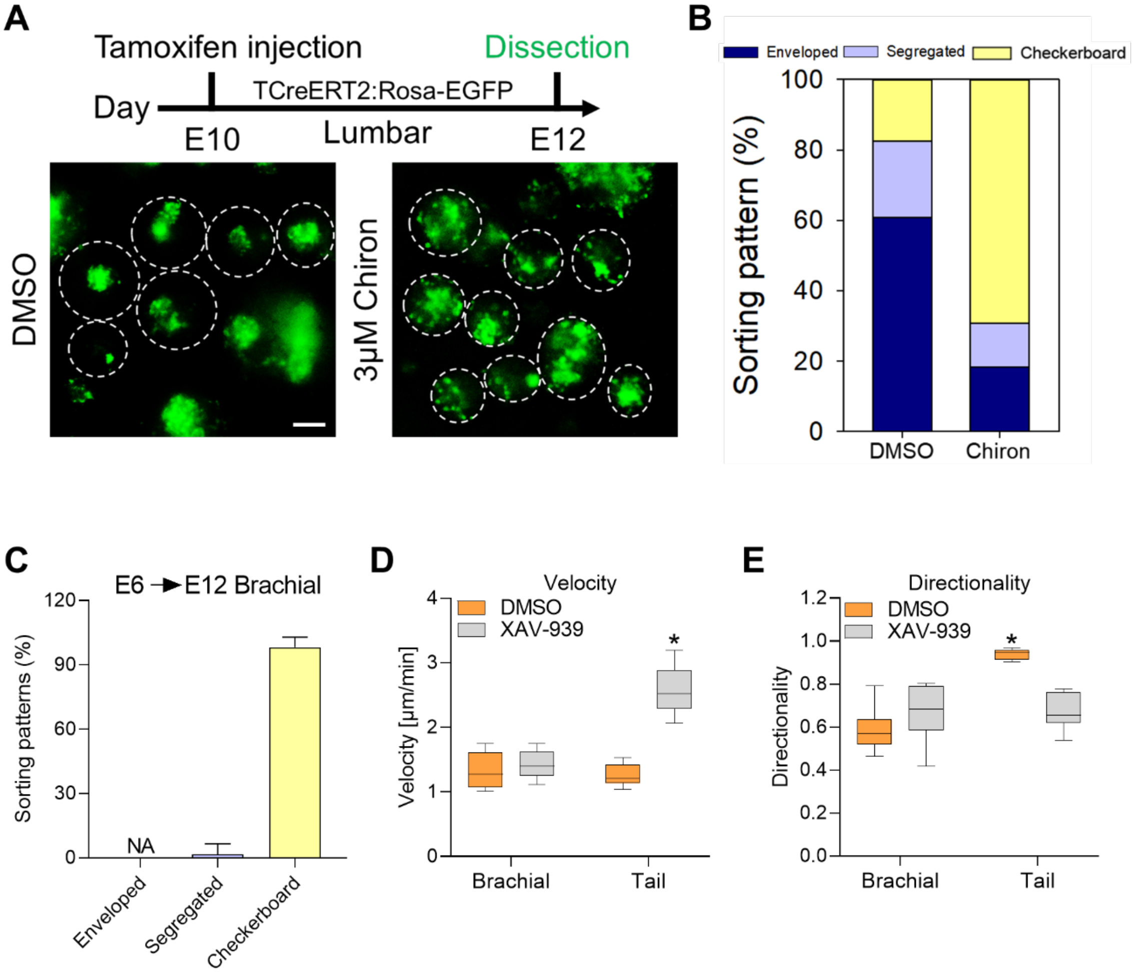

Figure S5. NPCs Adhesion Affinity Isolated from E12 TCreERT2:Rosa-EGFP Lumbar and Brachial tissue. (Related to Figure 2 and Figure 4)

(A) Neurospheres from TCreERT2:Rosa-EGFP embryos (E12) injected with tamoxifen at E10. White dotted lines show the margin of neurospheres. Scale bar is 100µm.

(B) Quantification of the percentage of sorting patterns upon Wnt activation with Chiron treatment; Number of independent experiments = 3; Number of examined neurospheres = 602; *P < 0.05 via One Way ANOVA.

(C) Quantifications of the percentage of sorting phenotypes in brachial-derived neurospheres. Brachial tissue treated with tamoxifen at E6 and dissected at E12 TCreERT2:Rosa-EGFP embryos. Data are shown as mean ± s.d; Number of independent experiments = 3; Number of examined neurospheres = 478; *P < 0.001 via One Way ANOVA. Abbreviations are E, Mouse Embryonic day. P, Passage.

(D) Box plots show the calculation of the velocity of neurospheres pretreated with either DMSO or XAV-939. Single measurement was obtained from the single migrating cell. Data are shown as median ± s.d.; Number of independent experiments = 3; *P < 0.001 via One Way ANOVA on ranks.

(E) Box plots show the calculation of the directionality of neurospheres pretreated with either DMSO or XAV-939. Single measurement was obtained from the single migrating cell. Data are shown as median ± s.d.; Number of independent experiments = 3; *P < 0.001 via One Way ANOVA on ranks.

Figure S6

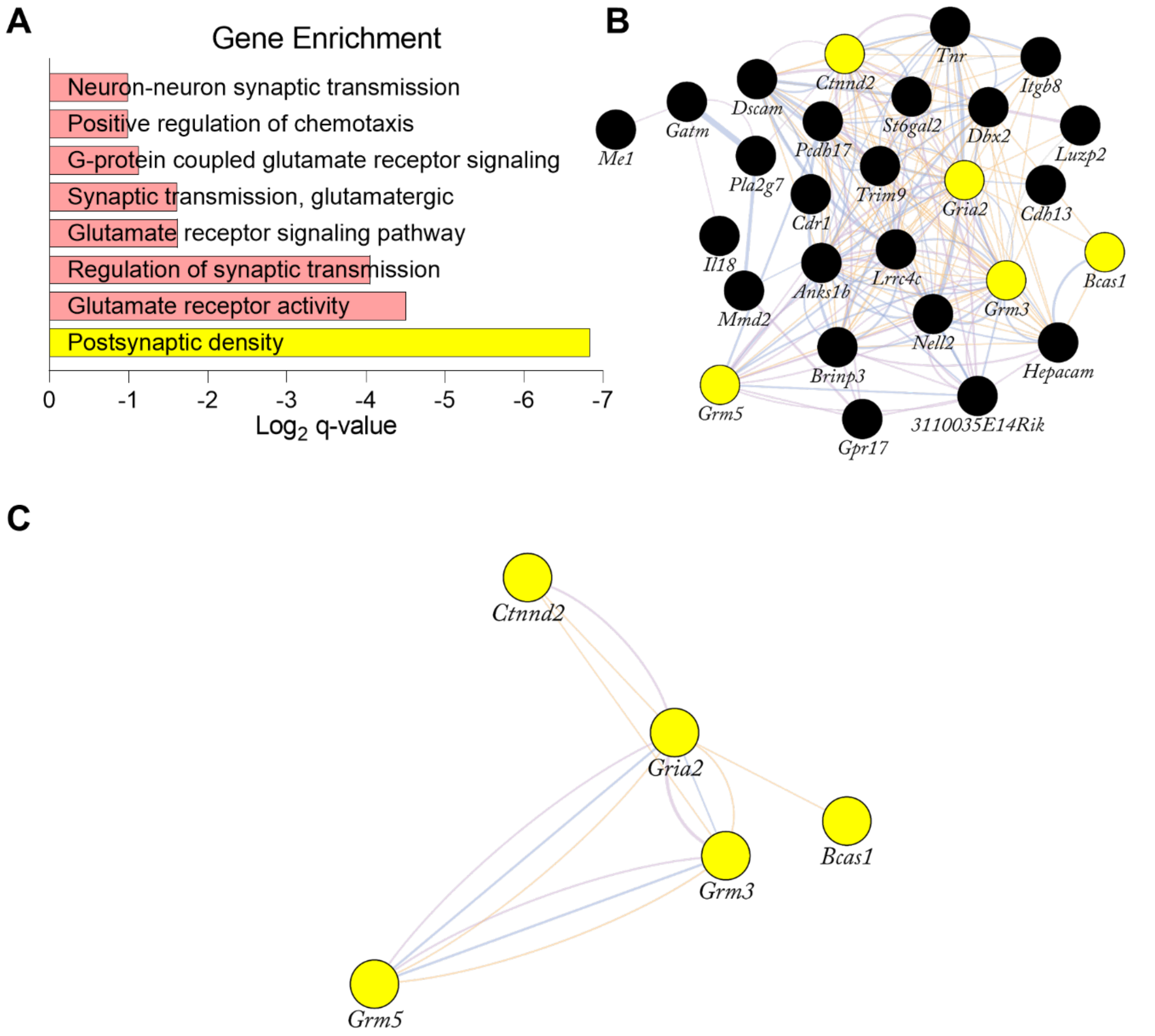

Figure S6. Illustration and Transcriptomic Analysis of Microarray. (Related to Figure 5)

(A) Bar graphs are presenting the enriched GO terms of top 10% down-regulated genes of tail-derived NPCs compared to brachial-derived NPCs. Yellow bar indicates the highest activation genes annotation group.

(B) The interaction network of top 10% down-regulated genes in tail-derived NPCs, yellow genes are grouped according to their GO analysis in (A), the interaction network was predicated using GeneMania and visualized in Cytoscape.

(C) Interaction network of genes that are enriched in postsynaptic density, the genes interactions were predicted using GeneMania and visualized in Cytoscape.

MULTIMEDIA FILE LEGENDS

Movie S1. Re-aggregation of rostral (GFP) and caudal (mCherry) hNPs demonstrating the enveloped phenotype. Cell re-aggregation was monitored using Juli™ stage equipped with objective lens 4× and placed in a 5% CO_2 incubator and 37°C. Images were taken per 10 min for 48 h.

Movie S2. Monitoring the migration mode of individual NPC derived from the tail and brachial levels after the scratch assay. Random and amoeboidal mode of brachial NPC migration is clearly observed in comparison to the collective migration of tail NPCs. Cell migrations were monitored using Juli™ stage equipped with objective lens 10× and placed in a 5% CO_2 incubator and 37°C. Images were taken per 10 min for 24 h.

Movie S3. Monitoring the migration mode of individual NPC derived from brachial level pretreated with Chiron for 4 days before the scratch assay. Adhesion between NPCs is enhanced upon Wnt activation with Chiron evident with a collective like migration phenotype. Cell migrations were monitored using Juli™ stage equipped with objective lens 10× and placed in a 5% CO_2 incubator and 37°C. Images were taken per 10 min for 24 h.

SUPPLEMENTAL TABLES

Table S1. List of Up and Down-Regulated Genes in Tail and Brachial-NPCs. Related to Figure 5. fc-Fold Change (Refer to the attached excel file).

Table S2. List of Up and Down-Regulated Genes in N_P and N_H NPCs. Related to Figure 5. fc-Fold Change (Refer to the attached excel file).

Table S3. List of Primer Sequences used for RT-PCR. Related to experimental procedures related to Figures 3-5

Gene	Primer (Forward)	Primer (Reverse)	Size
N-cadherin	GGCCAAGAAGGTACGTTTCTAC	AGCAACGCATCAAAACTACGAA	99
Ctnnb1	AGTGCAGGAGGCCGAGG	TCAAACTGCGTGGATGGGAT	427
Lef1	TCGACTTCAGGTGGTAAGAGA	GCTGGCTTTCTAGTTGCACG	447
Tcf4	GGCGATCTGAGGGGGAAAAT	CCGGAGGAACTTTTCGGACT	596
Axin2	GCTGCGCTTTGATAAGGTCC	TGGTCACCCAACAAGGAGTG	543
cyclin D1	GGTGGGCAGACCTCTTAACC	ACTTTGCAGGACAGATCCCG	544
Wnt1	GGTGGGGCATCGTGAACATA	ATTTGCACTCTTGGCGCATC	558
Frizzled	AAGCAGCGAACGTTGCAAAT	AATCGGTCACGAGCACAACT	126
Lrp5	CCATTGTGTTGCACCCTGTG	TGCACCCTCCATTTCCATCC	450
Apc2	TGGACACGTTTTCGATGCAG	GCCTCAGTGCCATGAAGGAT	460
Apc	TGTGGATCACGAAACAGCCA	CTGCAGTCCCCCAAGTTCAT	571
c-Myc	GTTGGAAACCCCGCAGAC	AGGTCTCGTCGTCAGGATCG	401
Mmp7	TCTTGGGCAGAATGTTCCTGG	GTGCGAAGGCATGACCTAGA	481
Rar alpha	CAGACACGCAGACGGGTTG	GTGTTGGGCAGGAACTGCTA	595
Rxr beta	AGCCATCTTTGATCGGTCCC	AACCCTGCTAAGTGTTGCCA	501
Dcn	TCCACACCTGCAAACCCTTT	GTTCCTGGAGAGTTCTGGGC	566
Col3a1	GCGAGCGGCTGAGTTTTATG	GGGTCACCATTTCTCCCAGG	494
Col1a2	GTGAACGTGGTGAAGTTGGC	AGCAATACCTGGCAGACCAC	530
Col1a1	GTCTGCCCAGCAAACAAAGG	TGCTGGTCTAGGGAGCATCT	507
Postn	CTGCTTCAGGGAGACACACC	GCCTCCCCTGGAAAGGTAAC	488
Lum	TGAGAACGTCACAGACCTGC	CGAGTTTCCAGGTACCCCAC	493
Snail1	AGCCCAACTATAGCGAGCTG	GGGTACCAGGAGAGAGTCCC	155
Pax3	CCGGGGCAGAATTACCCAC	GCCGTTGATAAATACTCCTCCG	99
Bra-T	ACAACCACCGCTGGAAATATG	CTCTCACGATGTGAATCCGAG	231
HoxB2	TTGAATTTGAGAGGGAGATTGGGT	TTTGACCTGCCTTTCGGTGA	551
HoxA7	AAAACTGTTACCGGCCCACA	GTAACCTGTCCGCCCAAAGA	575
HoxB7	CGAACAAACTTCTTGCGCCT	GTTCCCGGTCCTGAGGTTTT	528
HoxC10	AGCGCTATAACCGTAACGCA	AGCTGAGGCGATTCCAGATG	508
HoxD10	CATTTCTGCCGAGGTCCCTT	AATCTTGACCTGCCTGTCGG	568
HoxD11	ACACCAAGTACCAGATCCGC	TCTAGCCCCATGGCCTAACT	532
HoxA13	ACAAGTACATGGACACCGCC	TCGTAGCGTATTCCCGTTCG	408
HoxD13	CTCTTCGTCGTCGTCGTCAT	TTCCCCGGATCCAAAAGTGG	400
Gapdh	CATCACTGCCACCCAGAAGACTG	ATGCCAGTGAGCTTCCCGTTCAG	153

SUPPLEMENTAL EXPERIMENTAL PROCEDURES

Mice and Embryos

For transgenic mice, the TCreERT2 transgenic mouse line used in this work was generated as described previously (Anderson et al., 2013). The TCreERT2 transgenic and Rosa-EGFP reporter mice were purchased from Jackson ImmunoResearch Laboratory. TCreERT2 males were crossed with Rosa-EGFP females to generate TCreERT2: Rosa-EGFP transgenic males, which have *EGFP* insertion in the *Bra-T* promoter, with successful Cre excision upon tamoxifen injection being indicated by EGFP expression in Bra-T-expressing tissues. Rosa-EGFP females were then back-crossed with TCreERT2: Rosa-EGFP males and plugged females were separated on the next day and labeled as embryonic day 1 (E1). Pregnant females were subjected to treatment with 0.175 mg/g tamoxifen. Tamoxifen injection time was at E8 or E10 for labeling Bra-T expressing cells before and after NT closure, respectively. Pregnant females were then sacrificed at E12 for dissecting out the embryos and processed for *in vitro* and *in vivo* experiments. For experiments with wild-type animals, pregnant female C57BL/6 mice at E10 and E12 were purchased from Dae Han Biolink Co. Ltd (Korea). All experiments were performed in accordance with the ethical guidelines of Korea University and with the approval of the Animal Care and Use Committee of Korea University (KUIACUC-2015-22).

Embryonic Neural Progenitor Culture

Embryonic NPC culture was performed as described previously (Shaker et al., 2015). For tail-derived NPCs, somite tissue was removed as much as possible to prevent contamination of other cell types. For spinal cord-derived NPCs, tissues between forelimbs and hindbrain, forelimbs and hind-limbs, and between hind-limbs and tail were precisely dissected and grouped as brachial, thoracic, and lumbar, respectively. Dissected tissues were then incubated for 15 min with Accutase (Innovative Cell Technologies) at 37°C for complete dissociation of tissues into single cells. NPCs (10^4 cells) were seeded on ultra-low attachment surface culture dish (Corning: REF3471) and expanded into neurospheres for four days in N2 media containing supplements of 1% serum-free N2 (Gibco) and 2% B27 (Gibco), and 1% penicillin-streptomycin (Gibco BRL) in Dulbecco's modified Eagle medium (DMEM)/F12 (Welgene) with daily addition of bFGF (20 ng/ml; R & D) and EGF (20 ng/ml; Invitrogen). For viral infection, the virus concentration was adjusted to 1×10^9 transducing units per milliliter and GFP or RFP was injected into NPCs at day 1 as described previously (Kim et al., 2015b). The EVOS FL cell imaging system (Life Technologies) was used to assess ~~the~~ transfection efficiency. Four days after viral transfection, labeled-neurospheres were dissociated and single cells were used for the sorting assay. For various treatments, the following were added to N2 media containing EGF/bFGF: 3 μM Chiron (Sigma Aldrich) for activating the Wnt/β-

catenin signaling pathway, 200 ng/ml DKK-1 (Peprotech), 1 μM XAV-939 (Selleckchem), and 1 μM Wnt-C59 (Cellagen Technology) for blocking the Wnt/β-catenin signaling pathway, 1 μM RA for activating the RA signaling pathway, 200 ng of FGF8b and 50 ng GDF11. For NPCs differentiation, NPCs were seeded onto coverslips coated with poly-L-ornithine (PLO) (Sigma-Aldrich) and laminin (5 μg/ml; Invitrogen) and maintained in N2 media for six days without EGF/bFGF. Transfection of NPCs with GFP or RFP-retrovirus was carried out as described previously (Kim et al., 2015b).

Migration Assays

Scratch assay of NPCs was performed as described previously (Liang et al., 2007). Briefly, 100,000 cells from Passage 1 NPCs were seeded and cultured onto pre-coated six-well culture dish (SPL Life Sciences: Ref32006) with PLO and laminin for four days to create a confluent monolayer. A straight-line scratch was then made using a 1 ml pipette tip, and cells were washed with new N2 media containing bFGF/EGF to remove debris. The culture dish was placed back in the incubator at 37°C for 48 h, and a series of images were acquired from similar fields to calculate the percentage of wound closure. To assess the polarity of NPCs during migration, cells were fixed at 12 h of migration with 4% paraformaldehyde (PFA) for 20 min at room temperature (RT), and only migrating cells near the edge of the scratch were analyzed with appropriate markers. For the *ex vivo* migration assay, explants of similar sized neural tissues were dissected out from pregnant dams at E12 in pre-chilled Hank's buffered salt solution (HBSS) (Gibco). Neural tissues were transferred onto a six-well culture dish pre-coated with PLO and laminin-containing N2 media with EGF/bFGF. The dish was then incubated at 37°C for 12 h. Images were then acquired to measure the migration length of cells away from the edge of explants. Neurosphere radial migration was performed as described previously (Kim et al., 2015a). All images were acquired using the EVOS FL cell imaging system (Life Technologies), and measurement and quantifications were performed using the ImageJ program (USA National Institutes of Health).

Cell Sorting Assay

GFP- and RFP-labeled neurospheres were dissociated using Accutase (Innovative Cell Technologies) at 37°C for 10 min. Single cells were then co-cultured in 1:1 ratio with final plating density of 5×10^5 cells/2 ml N2 medium supplemented with EGF/bFGF. De-mixed cells were co-cultured for four days before quantification of sorting phenotypes. For pre-treatment sorting assay, NPCs at passage 0 were pre-treated with either DMSO (Sigma Aldrich), 3 μM Chiron, 200 ng/ml DKK-1 (Peprotech), 1 μM XAC-939 (Selleckchem), 1 μM Wnt-C59 (Cellagen Technology) or 1 μM RA (Sigma Aldrich) for four consecutive days. Pre-treated NPCs were then passaged and re-aggregated at passage 1 for four days to promote the formation of sorting phenotypes. Images of

live sorted neurospheres were then acquired using the EVOS FL cell imaging system (Life Technologies) to assess the percentage of sorting phenotypes.

Human Embryonic Stem Cells Culture and Differentiation

H9-GFP and H9-mCherry human embryonic stem cell (hESC) lines (Gift from Prof. Dong Wook Han from Konkuk University) were maintained in E8 medium (Stem Cell Technologies) on Matrigel (BD Bioscience). To initiate the differentiation, hESC clumps were passaged with ReLeSR (Stem Cell Technologies) onto Matrigel-coated plates in mTeSR medium (Stem Cell Technologies) to increase the efficiency of differentiation. The following day, ReLeSR was replaced with N2 media containing supplements of 1% serum-free N2 (Gibco), 2% B27 (Gibco), 1% penicillin-streptomycin (Gibco BRL), 1% modified Eagle medium (MEM) non-essential amino acids (Gibco) and 0.1% β-mercaptoethanol (Gibco) in DMEM/F12 medium (Welgene). Chiron (3 μM) (Sigma Aldrich) and 10 μM of SB 431542 (Tocris) were added for three days to induce NMPs, or dual Smad inhibitors 10 μM LDN-193189 (Stemgent) and 10 μM SB were added for three days to induce anterior NPCs. Both anterior and posterior NPC colonies were maintained in N2 media containing bFGF (25 ng/ml) for three days before the cell sorting assay. Sorting phenotypes of sorted human neurospheres were evaluated using the EVOS FL cell imaging system (Life Technologies).

Immunostaining

Immunohistochemistry (IHC) was performed as described in (Shaker et al., 2015), and immunocytochemistry was performed as described previously (Kim et al., 2015a). Whole-mount IHC for embryonic tails and the neurospheres was performed as described previously (Yokomizo et al., 2012). Embryonic tissues were fixed in 4% PFA for 20 min on ice, followed by three times washing with PBS at 4°C. Samples were then dehydrated in 100% methanol for 10 min at 4°C before incubation with DMSO:H_2O_2:Methanol 3:2:1 ratio overnight at RT. Samples were then washed three times with PBS before blocking with 10% BSA contains 5% DMSO to promote the penetration of antibodies. Neurospheres were fixed in 4% PFA for 20 min at RT, and the fixed neurospheres were then washed twice with 0.1× phosphate buffer saline Triton X-100 (PBST) for 10 min at RT. Bovine serum albumin (BSA) (6%) (Millipore) in 1× PBS buffer containing 0.2% Triton X-100 was used to block neurospheres for 12 h at RT.

Primary antibodies GFP (Abcam ab13970, 1:2000), BRA-T (R&D AF2085, 1:500), SOX2 (Millipore AB5603, 1:500), NESTIN (Millipore MAB353, 1:500), N-CADHERIN (BD Biosciences 610921, 1:500), Γ-TUBULIN (Santa Cruz j1612, 1:55), PAN-CADHERIN (Santa Cruz sc-8066, 1:500), Β-ACTIN (Sigma A5441, 1:500), GFAP (Invitrogen A12379, 1:500), TUJ1 (Sigma T2200, 1:1000), BETA-CATENIN (BD 610153, 1:500), BETA-

CATENIN (Sigma C2206, 1:500), TUJ1 (Millipore AB5320, 1:1000) and NG2 (Millipore AB5320, 1:500) were applied and incubated for 48 h at RT, and the samples were then washed thrice with 0.1× PBST at RT and incubated with secondary antibodies for an additional 48 h at RT. After washes with 0.1× PBST, the samples were mounted and imaged by confocal microscopy (Lecia TCS SP5). Alexa-488, Alexa-546, and Alexa-633-conjugated secondary antibodies were obtained from Jackson ImmunoResearch Laboratory.

Live Imaging

Live cell imaging analysis was performed as previously described (Kim et al., 2015a). Neurospheres of similar sizes and single cells were seeded on culture plates coated with PLO and laminin in N2 media containing EGF/bFGF. Neurospheres were allowed to migrate for 6 h, and the scratched monolayer cells were allowed to migrate till complete wound coverage was achieved. Images were acquired after every 10 min (interval time) using Juli^{TM} stage (NanoEntek, Seoul, Korea) under the 10× objective. The behavior of individual migrated cells was then analyzed using the ImageJ software (USA National Institutes of Health), and raw data were plugged in the Chemotaxis and Migration Tool software version 1.01 (Ibidi) to quantify directionality and velocity and to generate the migrating behavior video and cell trajectory of individual cells.

Imaging, Quantification, and Analysis

Acquired digital images were processed in Adobe Photoshop CS6. For calculating the migration distance of neurospheres and explants, total distance from the core to the edge of the radially migrated cells was measured using the ImageJ software (USA National Institutes of Health). Neurospheres and explants with similar sizes (ranged 180–210 µm) were included in the measurements. For the scratch assay, three images were acquired from the field along with the scratch on day 0, and images from similar fields were taken with 12 h interval time to calculate wound closure speed. ImageJ was used to stack images and generate a video of cell migration behavior. Images were acquired using the EVOS FL cell imaging system (Life Technologies), objective lens LPlan 2×/0.06. Matlab software (R2018a) was used to code and generate angle histogram (rose polar plot), the code being

```
sample = [angle measurement]
sample = sample./180*pi
sample = polarhistogram(sample,'Normalization','Probability')
```

The front to rear ratio was calculated from the polar plot of each group accordingly. For area measurement, scale bar was adjusted, and the area was quantified using Polygon in ImageJ. For aspect ratio, total width and length of the individual cell was measured, and the following formulas were then used to generate the aspect ratio values, the formula being

Aspect ratio = width/length

qRT-PCR

Total RNA was isolated from neurospheres as described previously (Shaker et al., 2015). For qPCR, SYBR Green with low ROX (Enzynomics) was used. PCR standard reaction conditions were set according to the manufacturer's instructions. PCR primers were designed using the NCBI free online system. All experiments were performed in biological duplicates or triplicates for each sample analyzed. Expression values were normalized against the GAPDH expression value of each sample, means and standard deviations were calculated and plotted using the Sigma Plot 12.5® software. Primers are listed in Appendix Table S3.

Western Blot

NPCs were collected and lysed with protein lysis buffer (100 mM Tris, pH6.8 and 4% SDS, and a cocktail of protease and phosphatase inhibitors (Roche)). After sonication of cells, protein concentration was quantified using bicinchoninic acid (BCA) protein assay kit according to the manufacturer's instructions. After quantification, samples were then heated for 10 min at 100°C. Optimized amount of protein was loaded and separated using 10% SDS-PAGE, separated proteins were then transferred onto a Nitrocellulose Blotting Membrane (Amersham™ Proton™ 0.2 µM NC). The membrane was then blocked by 5% BSA in TBS-T (20 mM Tris-HCl, pH 7.6, 136 mM NaCl, and 0.1% Tween-20) for 1h at RT, followed by primary antibody incubation for 12h at 4°C. The membrane was then washed with 1X TBST 3 times for 10 minutes each at RT before incubation with secondary antibody diluted 1:5000 in 5% Skim Milk in 1X TBST for 1h at RT. The membrane was washed again washed with 1X TBST 3 times for 10 minutes each at RT before visualization with ECL kit (Thermo, Cat# 32106).

Atomic Force Microscopy

For *in vivo* samples, brachial and tail tissues were dissected at E12 and sectioned into 1 mm slices before stiffness measurement. For *in vitro* samples, neurospheres were expanded as mentioned above, and similarly sized neurospheres were selected for stiffness measurement. All samples were measured by a Park's System's (NX10, Korea) instrument using the PinPoint™ nanomechanical mode at RT. The AFM cantilever used for measurement was a PPP-LFMR tip from Nanosensors Inc. with 10 nm R tip curvature, a silicon cantilever used for lateral/friction force microscopy. The measured signals were then converted to kPa unit using the Hertzian model, with Poisson's ratio of the sample value being 0.5.

Scanning Electron Microscopy

Expanded neurospheres were immediately fixed with 2.5% glutaraldehyde in 0.1M phosphate buffer at 4°C for 2 h, before washing twice or thrice with 0.1 M phosphate buffer. Fixed neurospheres were then soaked in 1% osmium tetroxide in 0.1 M phosphate buffer for 1 h 30 min at RT as a secondary fixation. Subsequently, fixed

neurospheres were dehydrated using a series of ethanol washes (60%, 70%, 80%, 90%, and 95%) for 15 min each, followed by three washes with 100% ethanol for 30 min each at RT. Dehydrated neurospheres were immersed twice in tert-butyl alcohol for 20 min at RT. Neurospheres were then frozen at –70°C and freeze-dried to remove tert-butyl alcohol. Finally, neurospheres were mounted on top of a sample holder with a carbon tape, coated with platinum, and viewed under a scanning electron microscope (Hitachi S-4700).

Microarray Data Acquisition and Analysis

Mouse embryonic NPCs samples were prepared as explained above. RNA purity and integrity were evaluated by ND-1000 Spectrophotometer (NanoDrop, Wilmington, USA), Agilent 2100 Bioanalyzer (Agilent Technologies, Palo Alto, USA). The Affymetrix Whole transcript Expression array process was executed according to the manufacturer's protocol (GeneChip Whole Transcript PLUS reagent Kit). cDNA was synthesized using the GeneChip WT (Whole Transcript) Amplification kit as described by the manufacturer. The sense cDNA was then fragmented and biotin-labeled with TdT (terminal deoxynucleotidyl transferase) using the GeneChip WT Terminal Labeling kit. Approximately 5.5 µg of labeled DNA target was hybridized to the Affymetrix GeneChip Human 2.0 ST Array at 45°C for 16 hours. Hybridized arrays were washed and stained on a GeneChip Fluidics Station 450 and scanned on a GCS3000 Scanner (Affymetrix). Signal values were computed using the Affymetrix® GeneChip™ Command Console software. Obtained raw data were extracted automatically in Affymetrix data extraction protocol using the software provided by Affymetrix GeneChip® Command Console® Software (AGCC). After importing CEL files, the data were summarized and normalized with the robust multi-average (RMA) method implemented in Affymetrix® Expression Console™ Software (EC). We exported the result with gene level RMA analysis and performed the differentially expressed gene (DEG) analysis. The comparative analysis between tail-NPCs and brachial-NPCs was carried out using fold change. For a DEG set, hierarchical cluster analysis was performed using complete linkage and Euclidean distance as a measure of similarity. Gene-enrichment and functional annotation analysis for significant gene list were performed based on Gene Ontology (www.geneontology.org/) using GeneMania and visualized using Cytoscape software 3.7.0®. Raw data can be accessed through the accession number GSE132089.

The RNA-seq data of N_H and N_P like NPCs were obtained from a previously published study (Gouti et al., 2014). N_P/ N_H ratio was done using Ingenuity® Pathway Analysis (IPA®) software. Genes with ≥2-fold change were subjected to GO analysis to obtained enriched gene ontology using GeneMania visualized using Cytoscape software 3.7.0®.

Statistical Analysis

Normally distributed data were expressed as the mean ± standard deviation of the mean of independent experiments. The median ± standard deviation was used to express the non-normally distributed data. The sample size was determined using power analysis. The number of biological replicates as well as the sample size are indicated in the figure legends. When comparing two groups, we used the Student's t-test. A one-way or two-way ANOVA was used for comparison of multiple groups, followed by the Tukey's post-hoc analysis for comparisons to a single control. Statistical analysis was performed using Sigma Plot 12.5® software. Minimal statistical significance was defined at $P < 0.05$.

SUPPLEMENTAL REFERENCES

Anderson, M.J., Naiche, L., Wilson, C.P., Elder, C., Swing, D.A., and Lewandoski, M. (2013). TCreERT2, a transgenic mouse line for temporal control of Cre-mediated recombination in lineages emerging from the primitive streak or tail bud. PloS one *8*, e62479.

Gouti, M., Tsakiridis, A., Wymeersch, F.J., Huang, Y., Kleinjung, J., Wilson, V., and Briscoe, J. (2014). In vitro generation of neuromesodermal progenitors reveals distinct roles for wnt signalling in the specification of spinal cord and paraxial mesoderm identity. PLoS Biol *12*, e1001937.

Kim, H.J., Shaker, M.R., Cho, B., Cho, H.M., Kim, H., Kim, J.Y., and Sun, W. (2015a). Dynamin-related protein 1 controls the migration and neuronal differentiation of subventricular zone-derived neural progenitor cells. Scientific reports *5*, 15962.

Kim, J.Y., Choi, K., Shaker, M.R., Lee, J.-H., Lee, B., Lee, E., Park, J.-Y., Lim, M.-S., Park, C.-H., Shin, K.S., *et al.* (2015b). Promotion of cortical neurogenesis from the neural stem cells in the adult mouse subcallosal zone. Stem cells *12*, 1-19.

Liang, C.-C., Park, A.Y., and Guan, J.-L. (2007). In vitro scratch assay: a convenient and inexpensive method for analysis of cell migration in vitro. Nature protocols *2*, 329-333.

Shaker, M.R., Kim, J.Y., Kim, H., and Sun, W. (2015). Identification and characterization of secondary neural tube-derived embryonic neural stem cells in vitro. Stem cells and development *24*, 1171-1181.

Yokomizo, T., Yamada-Inagawa, T., Yzaguirre, A.D., Chen, M.J., Speck, N.A., and Dzierzak, E. (2012). Whole-mount three-dimensional imaging of internally localized immunostained cells within mouse embryos. Nature protocols *7*, 421-431.
